# MIA40 circumvents the folding constraints imposed by TRIAP1 function

**DOI:** 10.1016/j.jbc.2025.108268

**Published:** 2025-02-03

**Authors:** Jordi Pujols, Marc Fornt-Suñé, Marcos Gil-García, Andrea Bartolomé-Nafría, Francesc Canals, Linda Cerofolini, Kaare Teilum, Lucia Banci, Sebastián A. Esperante, Salvador Ventura

**Affiliations:** 1 Institut de Biotecnologia i de Biomedicina and Departament de Bioquímica i de Biologia Molecular, Universitat Autònoma de Barcelona, Bellaterra, Barcelona, Spain; 2 Institut d’Oncologia Vall d’Hebron (VHIO), Cellex Center, Barcelona, Spain; 3 Magnetic Resonance Center (CERM), University of Florence, Sesto Fiorentino, FI, Italy; 4 Department of Chemistry "Ugo Schiff", University of Florence, Sesto Fiorentino, FI, Italy; 5 CIRMMP, Consorzio Interuniversitario Risonanze Magnetiche di Metalloproteine, Sesto Fiorentino, FI, Italy; 6 Structural Biology and NMR Laboratory, Linderstrøm-Lang Centre for Protein Science, Department of Biology, University of Copenhagen, Copenhagen, Denmark; 7 Hospital Universitari Parc Taulí, Institut d’Investigació i Innovació Parc Taulí (I3PT-CERCA), Universitat Autònoma de Barcelona, Sabadell, Spain

**Keywords:** oxidative folding, folding intermediates, TRIAP1, MIA40 pathway, molten globule, disorder-to-order transition

## Abstract

The MIA40 relay system mediates the import of small cysteine-rich proteins into the intermembrane mitochondrial space (IMS). MIA40 substrates are synthesized in the cytosol and assumed to be disordered in their reduced state in this compartment. As they cross the outer mitochondrial membrane, MIA40 promotes the oxidation of critical native disulfides to facilitate folding, trapping functional species in the IMS. Here, we study the redox-controled folding of TRIAP1, a small cysteine-rich protein with moonlighting function: regulating phospholipid trafficking between mitochondrial membranes in the IMS and preventing apoptosis in the cytosol. TRIAP1 dysregulation is connected to oncogenesis. Although TRIAP1 contains a canonical twin CX9C motif, its sequence characteristics and folding pathway deviate from typical MIA40 substrates. In its reduced state, TRIAP1 rapidly populates a hydrophobic collapsed, alpha-helical, and marginally stable molten globule. This intermediate biases oxidative folding towards a non-native Cys37-Cys47 kinetic trap, slowing the reaction. MIA40 accelerates TRIAP1 folding rate by 30-fold, bypassing the formation of this folding trap. MIA40 drives the oxidation of the inner disulfide bond Cys18-Cys37, and subsequently, it can catalyze the formation of the outer disulfide bond Cys8-Cys47 to attain the native two-disulfide-bridged structure. We demonstrate that, unlike most MIA40 substrates, TRIAP1's folding pathway is strongly constrained by the structural requirements for its function in phospholipid traffic at the IMS. The obligatory population of a reduced, alpha-helical, metastable molten globule in the cytoplasm may explain TRIAP1’s connection to the p53-dependent cell survival pathway, constituting a remarkable example of a functional molten globule state.

Since the initial description of the oxidative folding pathways of ribonuclease A and BPTI (1, 2), many small disulfide-rich proteins have been investigated to determine the molecular determinants that guide protein folding and native disulfide bond formation (3, 4) The slow kinetics of disulfide bond formation, together with the particular thiol chemistry, allowed the isolation and characterization of the major intermediates that populate the oxidative folding pathway of different proteins (5, 6). The spontaneous formation of native disulfide bonds is slow because it depends on adequate cysteine spatial positioning and requires an efficient final electron acceptor. Therefore, a dedicated oxidative folding machinery has been evolved to assist this process *in vivo*. In eukaryotic cells, disulfide bond formation primarily occurs in two compartments: the endoplasmic reticulum, catalyzed by the protein disulfide isomerase family and Ero1p (7, 8, 9), and in the mitochondrial intermembrane space (IMS) by oxidoreductase chaperones, such as MIA40 (10, 11).

TP53-regulated inhibitor of apoptosis (TRIAP1) is a small disulfide-rich protein that targets the IMS and participates in phospholipid trafficking between mitochondrial membranes (12). Its native structure consists of an antiparallel helix-loop-helix connected by two intramolecular disulfides, referred to as the inner disulfide (Cys18-Cys37) and the outer disulfide (Cys8-Cys47), followed by a disordered C-terminal region (Fig. 1) (13). Both α-helices are remarkably amphipathic and, as a consequence of the disulfide bridging, their hydrophobic faces become juxtaposed and partially exposed to solvent, which endorses the protein surface with an unusual nonpolar character. In the IMS, TRIAP1 binds to distinct PRELI domain-containing proteins, termed PRELID1, PRELID2, SLMO1, and SLMO2, to form heterodimeric complexes able to extract and transfer specific lipids from the outer membrane of the mitochondria to the inner membrane of the mitochondria (14). Binding to TRIAP1 stabilizes the PRELI-like proteins, which are unstable and rapidly degraded in their free state. An exposed hydrophobic cleft in TRIAP1 delineates the interacting surface with PRELID-like proteins (13). PRELI-TRIAP1 heterodimer is responsible for the biogenesis and accumulation of cardiolipin (CL) and phosphatidylethanolamine in inner membrane of the mitochondria (15). Of note, the CL and phosphatidylethanolamine's asymmetric composition of mitochondrial membranes determines the organelle's morphology and function. Perturbing lipid homeostasis and unbalancing CL levels result in mitochondria dysfunction and the activation of apoptotic signaling pathways (16, 17).

**Figure 1:**
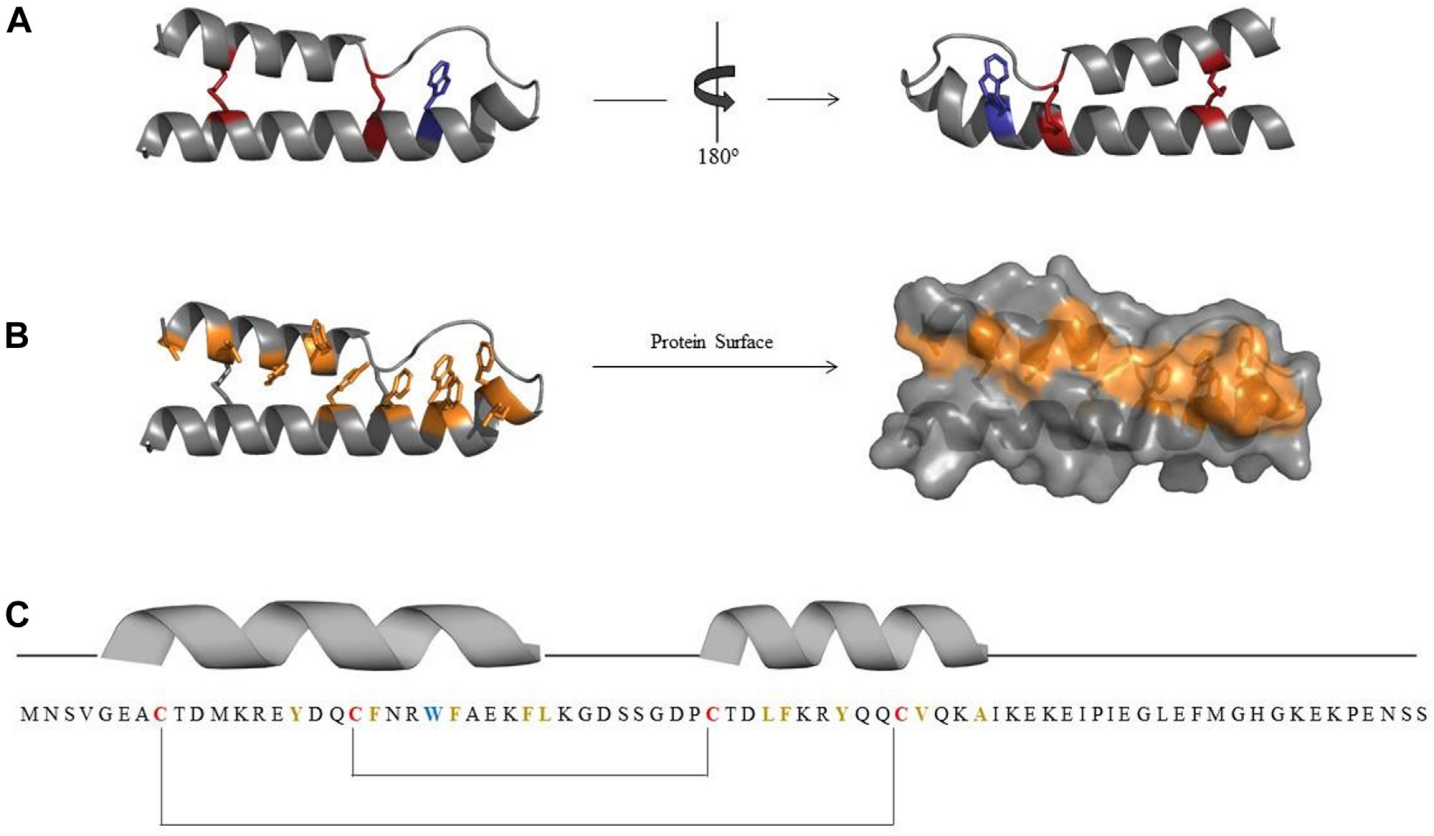
**Schematic view of the three-dimensional structure of TRIAP1.***A*, ribbon representation of TRIAP1. The cysteine residues and disulfide bonds (in *red*) and the tryptophan at position 22 (in *blue*) are indicated. *B*, surface representation of residues Y15, F19, W22, F23, F27, F41, and Y44 with their aromatic side chains forming a hydrophobic solvent-exposed surface. *C*, schematic representation of the primary structure of TRIAP1 with its secondary structure elements and disulfide pairing. The Protein Data Bank accession number for the structure of TRIAP1 is 4XZV. All figures were generated using PyMOL.

In the cytosol, TRIAP1 has been associated with the p53-dependent cell survival pathway by inhibiting the formation of the apoptosome complex of Apaf-1, cytochrome c, and caspase-9 proteins (18). In low DNA-damage cellular conditions, p53 upregulates the expression of TRIAP1 that, in complex with heat-shock protein 70 (HSP70), hijacks Apaf-1, thus acting as an anti-apoptotic agent. On the contrary, the uncontrolled and p53-independent overexpression of TRIAP1 correlates with the onset and malignancy of some types of cancers (19, 20). In this later case, TRIAP1 behaves as an oncogene, becoming an active molecular agent of pathogenesis.

TRIAP1, like most mitochondrial proteins, is synthesized in the cytosolic ribosomes. Therefore, it must be imported to the IMS to carry out its function in lipid homeostasis. The translocation of disulfide-rich proteins to the mitochondria relies on the MIA40 pathway, which couples import with oxidation and folding (21). TRIAP1 cysteines are encompassed in a highly conserved CX_9_C twin motif common to many MIA40 substrates. To be imported, disulfide-rich proteins must be in their reduced states. As they cross the mitochondria's outer membrane, MIA40 recognizes its substrates through hydrophobic interactions and introduces a mixed disulfide that pulls the polypeptide inside the IMS (22, 23, 24) This covalent interaction that results in a dynamic metastable complex between hMIA and its substrates is considered as the main driving force for efficient protein import and oxidative folding (24).

Therefore, MIA40 substrates are mostly reduced and disordered in the cytosol (25), and their premature oxidation, before reaching the IMS, competes with their efficient import (26). In this line of evidence, we recently reported that reduction and disorder are correlated for two different MIA40 substrates, Cox17 and Cox19 (27, 28). This connection is not limited to MIA40 substrates, but rather a generic feature of small disulfide-rich proteins, which, as a trend, resemble *bona fide* intrinsically disordered proteins (IDPs) in their reduced states (29). In this context, we hypothesized that TRIAP1 dual function in the cytosol and mitochondria stems from the population of two different redox and conformational states in these compartments.

In the present work, we exploit the slow and disulfide-limited folding of TRIAP1 to characterize the structural properties of its reduced state. We also study both the spontaneous and catalyzed oxidative folding reactions of TRIAP1 in the absence and presence of hMIA40. We found that TRIAP1 behavior differs significantly from other previously studied MIA40 substrates, including COX17 and COX19 (3, 27, 28, 30). Instead of being disordered, the TRIAP1 reduced state is collapsed and contains residual secondary structure elements, resembling a molten globule. The population of this metastable state impacts the oxidative folding of TRIAP1, by promoting the accumulation of a kinetically trapped off-pathway intermediate and slowing down the folding reaction. Remarkably, hMIA40 suffices to funnel the oxidative folding, fostering an efficient and fast reaction. These findings provide new structural and kinetic insights to understand the compartment-dependent function/malfunction of TRIAP1.

## Results

### TRIAP1 sequence intrinsic properties differ from that of canonic MIA40 substrates

**Figure 2:**
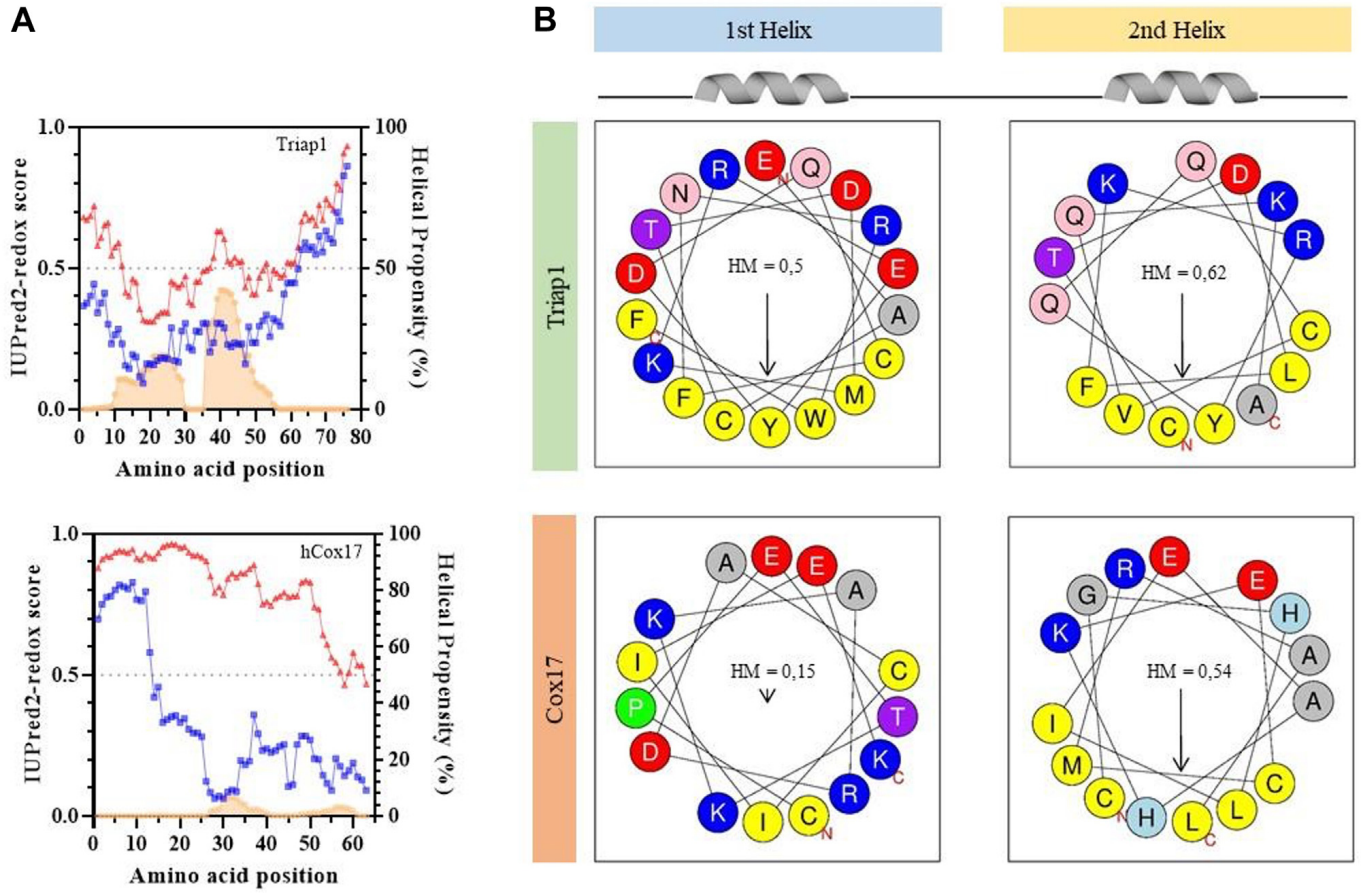
**Predictions on TRIAP1 disorder and ellipticity.***A*, disorder prediction of TRIAP1 and COX17 with the redox-sensitive algorithm IUPRED 2.0 (64). *Blue* lines correspond to the WT sequence, and *red* lines to the Cys to Ser versions that mimic the reduced state. Predictions of protein ellipticity provided by the AGADIR are plotted as *orange* areas (65). *B*, schematic representation of the amino acid spatial positioning for the first and second helices of TRIAP1 and COX17. Figures generated by the HeliQuest tool (66). The Hydrophobic Moment (HM) is indicated for each helix.

We have recently reported that COX17 and COX19, two CX_9_C substrates of hMIA40, are fully disordered in their reduced states. Upon oxidation, these proteins undergo a disorder to order transition (27, 28, 31). Since protein disorder is encoded in protein sequences, we first predicted the degree of disorder of TRIAP1 with the redox-sensitive algorithm IUPRED 2.0 and compared it with COX17 (Fig. 2*A*). In good agreement with their experimentally determined folded structures, the N- and C-terminus of COX17 and TRIAP1 are predicted as disordered in the oxidized state. However, while the reduced state of COX17 is predicted to be fully disordered (27), the reduced form of TRIAP1 does not possess continuous disordered regions. Conversely, TRIAP1 has a much higher helical propensity than COX17, as predicted by the AGADIR algorithm. When estimated from the average values of the helices in the respective helix-loop-helix domains, the helix propensity of TRIAP1 is three-fold higher for helix 1 and twenty-four-fold higher for helix 2 (10.5 and 28.5) than in COX17 (3.5 and 1.2) (Fig. 2*A*).

One common feature of MIA40 substrates is that they display an overall moderate hydrophobicity. Usually, one of the helices is significantly more hydrophobic since it incorporates the MISS/ITS signal that facilitates MIA40 recognition (11, 32). This helix is amphipathic, with the hydrophobic side becoming mostly protected from the solvent at the interface of the helix-loop-helix in the folded state. The other helix is less hydrophobic and has a low amphipathic character, as illustrated by COX17's first helix (Fig. 2*B*). Remarkably, both helices of TRIAP1 are highly amphipathic, with a particular enrichment in aromatic residues. Besides, this hydrophobicity remains significantly solvent exposed in the folded state, resulting in a total of 783.2 Å^2^ of hydrophobic exposed area, which is a 6-fold increase compared to the 124.0 Å^2^ of COX17. Since TRIAP1 helices are longer and comprise ten extra residues, the corrected value of hydrophobic-exposed area per residue is 19.1 Å^2^ for TRIAP1 and 4.0 Å^2^ for COX17 structures, still accounting for a > 4-fold increase.

Overall, the low propensity towards disorder, together with TRIAP1 inherent predicted ellipticity and hydrophobicity, indicates that TRIAP1 sequence intrinsic properties deviate from those of the canonic MIA40 substrates studied to date.

### TRIAP1 disulfide reduction leads to partially folded species

**Figure 3:**
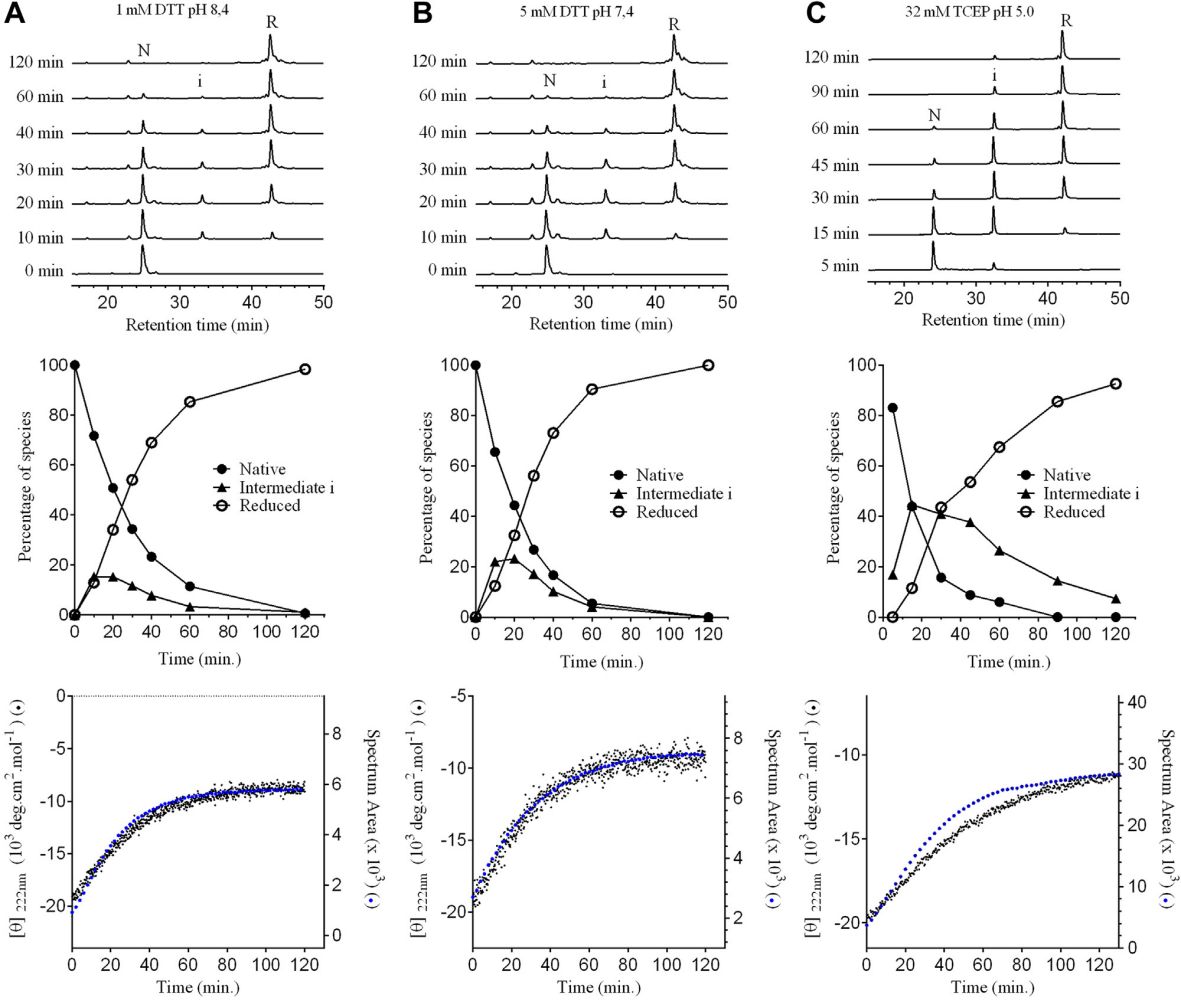
**Conformational changes upon disulfide reduction of TRIAP1.** Native TRIAP1 at 15 μM was treated with 1 mM DTT in 0.1 M Tris–HCl pH 8.4 (*A*), 5 mM DTT in 0.1 M sodium phosphate pH 7.4 (*B*), or 32 mM TCEP in 0.1 M sodium acetate pH 5.0 (*C*). *Upper panels*: After the addition of reducing agents, time course intermediates were trapped by acidification and analyzed by RP-HPLC. Native (N), intermediate (i), and reduced (R) TRIAP1 peaks are indicated. *Middle* panels: Percentage of species along folding time calculated as the area under the curve of RP-HPLC chromatograms shown in the *upper* panels; reduced (○), intermediate i (▲), and native (•) species. Three independent experiments yielded similar results. A representative experiment is shown. *Lower panels*: conformational changes upon disulfide reduction followed by changes in molar ellipticity at 222 nm (*left* Y-axis, *black dots*) or changes in the tryptophan spectrum area (*right* Y-axis, *blue dots*) over time.

Disulfide bonds stabilize the secondary and tertiary structure of disulfide-rich proteins, and cysteine reduction is often coupled to unfolding, a process known as reductive unfolding (33). The reductive unfolding reaction of TRIAP1 was studied by incubating the native protein in the presence of reducing reagents at pH values of 8.4, 7.4, and 5.0. The concentration of reducing reagents was selected to obtain the fully reduced protein within 2 h of reaction at RT. The unfolding reaction was trapped in a time-course manner by acidification and analyzed by RP-HPLC (Fig. 3). At pH values of 8.4 and 7.4, the unfolding reactions occurred with a low accumulation of a single intermediate species (i) that eluted at 32.5 min. Disulfide reduction using TCEP at pH 5.0 minimizes disulfide reshuffling, and in this condition, intermediate (i) accumulates to a greater extent (Fig. 3). Alkylation of purified i species with either iodoacetamide (IAA) or 2-vinyl pyridine and matrix-assisted laser desorption/ionization time-of-flight (MALDI-TOF) analysis revealed that it corresponds to a 2-cysteine free intermediate.

Reductive unfolding was also monitored by changes in the secondary and tertiary structure using far UV-CD and tryptophan fluorescence, respectively. TRIAP1 harbors a single tryptophan at position 22 and a high α-helical content in the native state (see Fig. 1) (13). Disulfide reduction led to an increase in tryptophan fluorescence and a decrease in the α-helix content. The secondary and tertiary structural changes were synchronous at pH values of 8.4 and 7.4, while both signals were uncoupled at pH 5.0, with the tertiary structure changes slightly preceding those in secondary structure (Fig. 3). As predicted, fully reduced TRIAP1 still contained a significant amount of residual secondary structure, suggesting that the α-helices were not fully unfolded upon reduction.

### Reduced TRIAP1 resembles a molten globule

**Figure 4:**
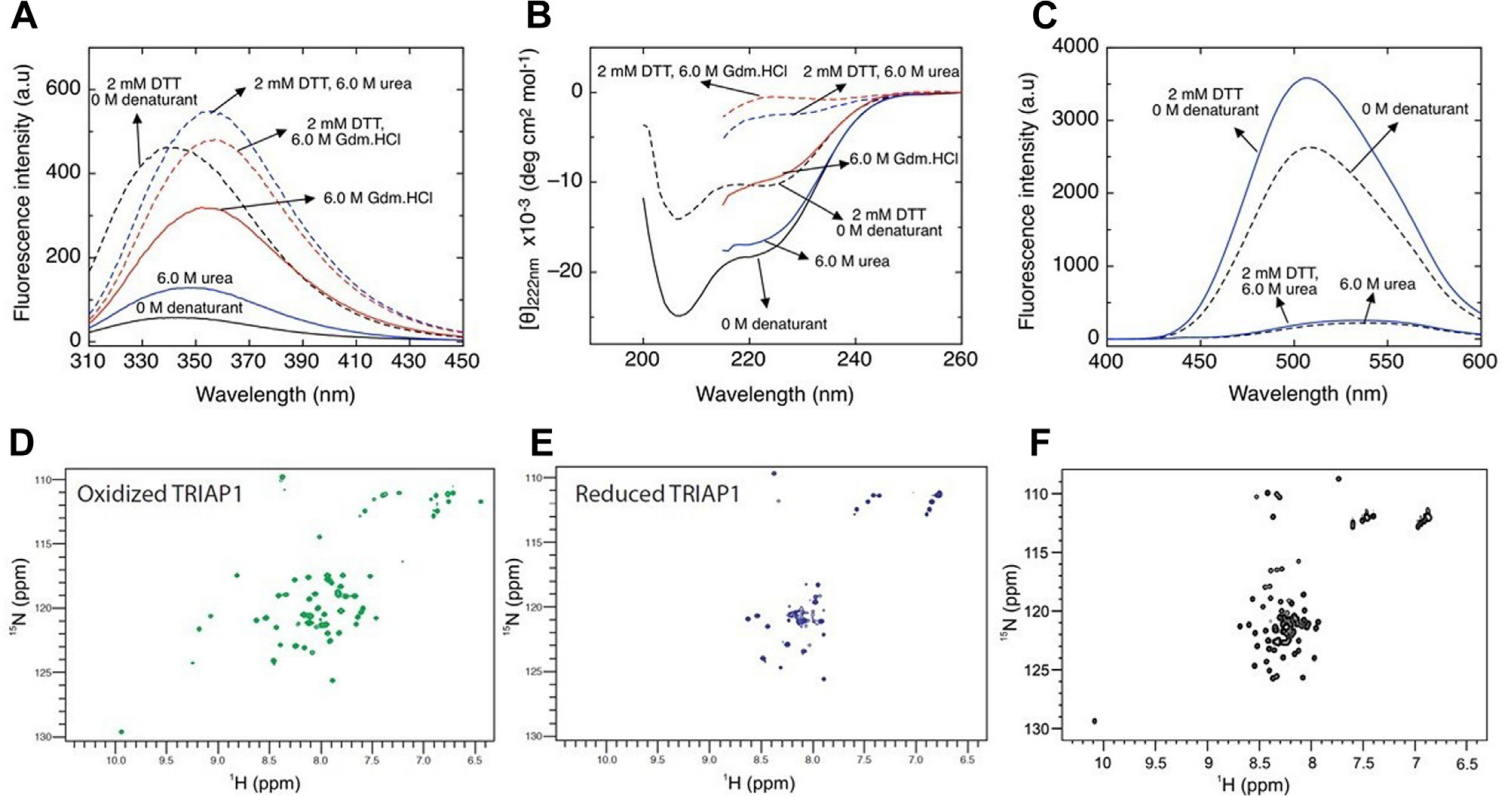
**Conformational properties of the fully reduced and fully reduced/unfolded TRIAP1.** Fifteen micromolars of TRIAP1 samples were incubated at RT for 20 h in 50 mM sodium phosphate pH 7.4 containing 0 M denaturant; 6.0 M urea or 6.0 M Gdm.HCl in the absence or presence of 2 mM DTT. After incubation, samples were analyzed by tryptophan fluorescence (*A*), far-UV CD (*B*), and binding to bis-ANS (*C*). The spectrum of each sample is indicated in the graph. After measurements, the samples were acid-quenched and subjected to RP-HPLC to confirm the fully reduced or oxidized state (see Fig. S1). 2D ^1^H ^15^N-HSQC NMR spectra of the oxidized (*D*) and reduced (*E*) TRIAP1 samples, respectively, at the concentration of 0.3 mM in 20 mM sodium phosphate pH 7.0 with 10% D_2_O. *F*, 2D ^1^H ^15^N-HSQC NMR spectra of reduced TRIAP1 in the absence (*red*) and presence (*black*) of 6 M Urea.

To further characterize both, the reduced and the reduced/unfolded states, native TRIAP1 samples were incubated for 16 h at pH 7.4 in the presence or absence of 2 mM DTT, either under nondenaturing conditions or adding 6.0 M urea or 6.0 M Gdm.HCl. The tryptophan fluorescence emission spectrum of native TRIAP1 in the absence of denaturant showed that its single tryptophan was partially exposed to the solvent with a maximum emission at wavelength of 336 nm (Fig. 4*A*). When the protein was reduced, the fluorescence intensity increased by 10-fold, indicating that the presence of the disulfide bonds quenches the tryptophan fluorescence, as described for other small disulfide-rich proteins (34). The fully reduced TRIAP1 exhibited a maximum emission at wavelength of 340 nm, indicating a similar tryptophan environment compared to the native disulfide-bridged protein. As described above, the far UV-CD spectrum of the reduced protein contained a significant amount of residual secondary structure characterized by a molar ellipticity at 222 nm of −10.400 deg.cm^2^.mol^−1^. This value corresponds to 27% of residues in helical conformation (35), compared to 40% for native TRIAP1, calculated from a molar ellipticity at 222 nm of -20.000 deg.cm^2^.mol^−1^ (Fig. 4*B*). The disulfide-oxidized TRIAP1 incubated in 6.0 M urea also displayed significant secondary structure and a tryptophan emission spectrum close to that of the native species. Incubation in 6.0 M Gdm·HCl, instead, partially unfolded TRIAP1, albeit still showing a substantial amount of α-helical content (Fig. 4*B*). The addition of 2 mM DTT, either in the presence of 6.0 M urea or 6.0 M Gdm·HCl, led to the complete loss of the secondary structure, with the tryptophan maximum emission wavelength shifted to 356 nm, indicating a complete exposure of the tryptophan to the solvent. These findings confirm that fully reduced TRIAP1, in the absence of denaturing agents, retains a significant amount of secondary and tertiary structures. After far-UV CD and fluorescence measurements, the samples were acid-quenched and subjected to RP-HPLC to confirm that TRIAP1 species were either fully oxidized or fully reduced, as intended (Fig. S1).

We next addressed whether TRIAP1 species contain hydrophobic patches exposed to the solvent, by monitoring their bis-ANS–binding ability (Fig. 4*C*). Under denaturing conditions, the protein did not show any significant bis-ANS fluorescence, both in the presence or absence of DTT. Remarkably, the reduced but not denatured TRIAP1 displayed increased bis-ANS fluorescence if compared to the oxidized and properly folded conformation. Thus, fully reduced TRIAP1 likely corresponds to a partially folded metastable structure with solvent-accessible packed hydrophobic residues.

To gain further insights into the conformations acquired by oxidized and reduced TRIAP1, the protein expressed isotopically enriched in ^15^N and 2D ^1^H ^15^N-HSQC NMR spectra in different oxidative and denaturing conditions were recorded. Under oxidizing conditions, the signals of amide protons are well dispersed, indicating that the protein is well-folded (Fig. 4*D*). After reduction of disulfide bonds, several of the well-spread cross-peaks disappear without the appearance of new signals. Indeed, in the spectrum of reduced TRIAP, the number of visible cross-peaks is less than expected, and they are primarily clustered in the region of the spectrum between 8 and 8.5 ppm, as occurs for disordered conformations (Fig. 4*E*). However, the presence of signals with different relative intensities and line broadenings suggests the coexistence of multiple conformations in solution. We could hypothesize that the protein under reducing conditions resembles an intermediate (molten globule) state where some parts of the protein are in conformational exchange on the microsecond-millisecond time scale, giving rise to extreme line broadening and peak merging in the NMR spectrum. This assumption was further confirmed by the 2D ^1^H-^15^N-HSQC NMR spectrum of TRIAP1 recorded in the presence of both DTT and 6.0 M urea (Fig. 4*F*). The combination of disulfide reduction and chemical denaturation results in a cleaner NMR spectrum, if compared to the spectrum of TRIAP1 in the presence of DTT only, showing well-defined and sharp cross-peaks in the region between 8 and 8.5 ppm, that correspond to a single species with a single conformation.

Finally, thermal denaturation of reduced TRIAP1 followed by far UV-CD revealed a gradual loss of its secondary structure upon heating, displaying low but still significant cooperativity (Fig. S2), confirming that reduced TRIAP1 is at least partially structured. As expected, when native disulfide cross-linked TRIAP1 was subjected to thermal denaturation analysis, it showed little changes in the secondary structure content as its fold is strongly stabilized by the covalent links (Fig. S2). Thermal denaturation of reduced TRIAP1 was fully reversible, and the spectrum of the reduced sample at 85 °C showed that the minimum at 208 nm, characteristic of α-helical conformation, shifted to lower wavelengths, indicative of an order to disorder transition upon thermal denaturation (Fig. S2).

Overall, the results of this section indicate that, in contrast to previously characterized MIA40 substrates, the reduced state of TRIAP1 resembles a molten globule conformation, characterized by the presence of secondary structure elements in a hydrophobic collapsed, but dynamic, state.

### Equilibrium denaturation of the molten globule state of reduced TRIAP1

**Figure 5:**
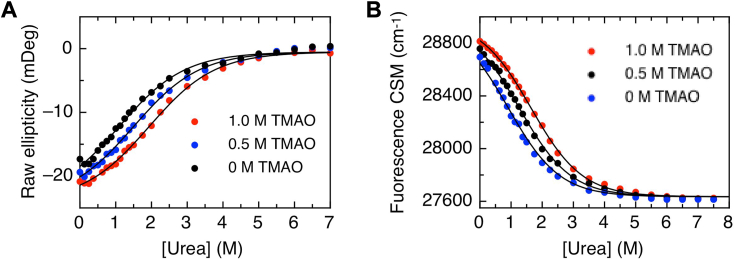
**Urea equilibrium denaturation of reduced TRIAP1 molten globule.** Urea equilibrium denaturation curves of 20 μM TRIAP1 were performed in the absence or presence of 0.5 or 1.0 M TMAO at 5 °C. Denaturation was followed by far-UV CD at 222 nm (*A*) and by tryptophan fluorescence CSM (*B*); solid lines represent fits of each curve to a two-state folding model (See Experimental procedures). Thermodynamic parameters obtained from the fits are summarized in Table 1. The experiment was performed twice, with similar results, a representative experiment is shown.

To quantitatively assess the folding/unfolding energetics of the molten globule state of reduced TRIAP1, we performed urea equilibrium denaturation in the presence of increasing concentrations of the stabilizing osmolyte trimethylamine oxide (TMAO) (36, 37). The unfolding process was followed by changes in the ellipticity at 222 nm or the tryptophan center of spectral mass (CSM). In the absence of TMAO, the unfolding curves lacked a pretransition region, while the addition of increasing concentrations of TMAO revealed a pretransition baseline (Fig. 5). As expected, increasing TMAO concentrations shifted the transition midpoints towards higher urea concentrations. Considering that TMAO stabilization of molten globule states takes place without changing the baselines of the unfolding curves or the *m*-value of the process (36), fluorescence and CD denaturation data were fitted individually to a two-state denaturation model (See Experimental procedures) (Fig. 5). Fitting results are summarized in Table 1. Comparing the CD and CSM unfolding curves at each TMAO concentration, the signals were similar but not overlapped completely, revealing that the loss of tertiary structure slightly precedes helix unfolding (Fig. S3). In the absence of TMAO, the free energy of unfolding (ΔG^0^_H2O_) for the molten globule state at 273.15 K was -0.65 ± 0.02 kcal/mol or -0.612 ± 0.01 kcal/mol, estimated from the CD and fluorescence CSM data, respectively. Based on the linear correlation of *m* value with the change in solvent-accessible surface area upon unfolding (dASA) (38), an average *m* value of 634.6 cal/mol.M^−1^ corresponds to the exposure of 2369 Å^2^, which represents the complete unfolding of 35 residues of TRIAP1 out of 76.

**Table 1:** Thermodynamic parameter estimates from the CD denaturation data (left side) and fluorescence denaturation data (right side) in the presence of increasing concentrations of TMAO

Thermodynamic parameters	CD denaturation data			Fluorescence denaturation data		
TMAO	1.0 M	0.5 M	0 M	1.0 M	0.5 M	0 M
ΔG(Kcal/mol)	1.15 ± 0.07	0.89 ± 0.01	0.65 ± 0.01	1.1 ± 0.04	0.85 ± 0.01	0.61 ± 0.01
m(kcal/mol/M)	0.6 ± 0.03			0.67 ± 0.02		

Overall, the collected data strongly suggest that the molten globule state of reduced TRIAP1 consists of a marginal stable ensemble of alpha-helical rich, hydrophobic-collapsed interconverting structures.

### TRIAP1 air-oxidizes to spontaneously attain its native folded state

**Figure 6:**
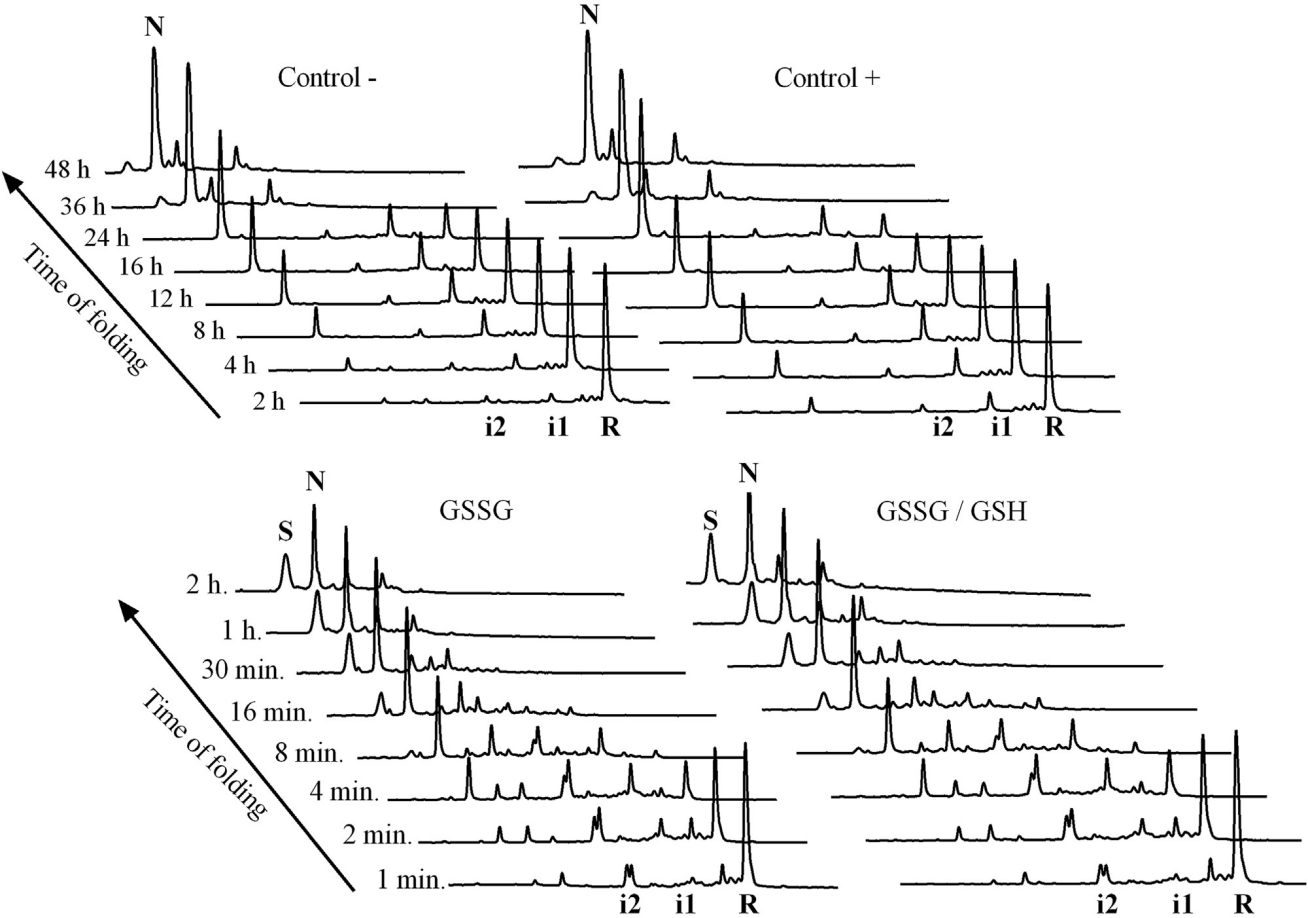
**RP-HPLC analysis of TRIAP1 refolding intermediates.***In vitro* refolding was carried out in 0.1 M Tris.HCl (pH 8.4) in the absence (Control -) or presence of selected redox agents: 0.25 mM β-mercaptoethanol (Control +), 0.5 mM GSSG or a mixture of 0.5 mM GSSG, and 1.0 mM GSH. Intermediates were acid-trapped at the noted times and analyzed by RP-HPLC. The native (N), reduced (R), scrambled species (S), as well as the two major intermediates (i1 and i2) are indicated. A detailed comparison of RP-HPLC chromatographic profiles is shown in Fig. S4.

We have previously demonstrated that MIA40 substrates can fold in the absence of oxidoreductases by air-oxidization (27, 28). Purified TRIAP1 was fully reduced and unfolded, subjected to buffer exchange, and allowed to refold at pH 8.4 in the presence of different redox agents (See Experimental procedures). Folding intermediates were acid-trapped at selected time points and subjected to RP-HPLC. The heterogeneity and chromatographic behavior of folding intermediates are shown in Figures 6 and S4. In the absence of redox reagents (Control -), there was a low accumulation of two folding intermediates: the first one at 37.5 min (hereafter i1), accumulated to a greater extent and showed a retention time close to the reduced species (42 min). The second heterogeneous intermediate peak (hereafter i2) eluted at 32.5 min, a similar retention time to that of the homogenous intermediate i peak populated in the reductive unfolding reaction (Figs. 3*C* and 6, S4, and S5). The refolding reaction was almost completed after 24 h incubation. At both 36 h and 48 h incubation, native TRIAP1 was in equilibrium with three other minor species, not found at early stages (Fig. 6). These species were not detected by RP-HPLC, neither in freshly purified native TRIAP1 nor when TRIAP1 was incubated for 24 h at pH 7.4. We speculate that these peaks likely correspond to off-pathway scrambled species. The presence of 0.25 mM 2-mercaptoethanol (Control +), which promotes disulfide rearrangement in oxidized species, neither affected the overall chromatographic pattern nor accelerated the folding reaction, indicating that disulfide isomerization was not a critical step in the formation of native disulfide bridges. Oxidative folding of TRIAP1 was subsequently performed in the presence of either oxidized glutathione (GSSG) or of a mixture of reduced and oxidized glutathione (GSH:GSSG). Both redox conditions accelerated the folding reaction, which was almost completed after 15 min (Fig. 6). The RP-HPLC chromatographic patterns obtained were similar, and the folding reaction progressed at similar rates. Intermediates i1 and i2 were also present, together with the population of other minor species and, in contrast to the folding reaction of Control -, intermediate i2 accumulated to a greater extent. Noteworthy, intermediate peak i2 displayed a heterogeneous profile containing at least two species with similar retention times (Fig. S5). The acceleration promoted by GSSG seems to stem at a greater extent from stochastic oxidation with the population of species that are not necessarily productive. After 1 and 2 h of refolding, a peak with a lower retention time than the native peak was accumulated, which might correspond to a compact scrambled species exposing less hydrophobic surfaces than the natively cross-linked form (See Figs. 6 and S4).

The secondary and tertiary structural changes of TRIAP1 along the folding process (Control -) were monitored by far-UV CD and tryptophan fluorescence emission spectra, respectively. The changes in both spectroscopic signals overlapped along time indicating that the secondary and tertiary structure formation upon folding are coupled (Fig. S6).

### Disulfide-pairing analysis of TRIAP1 folding intermediates i, i1, and i2

To gain insight into the disulfide connectivity, intermediates i, i1, and i2 together with native and fully reduced TRIAP1 were purified by RP-HPLC, freeze-dried, and subjected to a sequential alkylation-reduction-alkylation strategy, as described in Experimental procedures. As a first step, 2-vinylpyridine labeled the free cysteines and, after a disulfide reduction step, IAA labeled cysteines that were initially protected by the formation of disulfide bonds. The double derivatized intermediates were subsequently digested with trypsin and subjected to MALDI-TOF analysis. The molecular masses of the major derivatized peptides, which allow the identification of intermediates, are summarized in Table 2. The homogenous acid-trapped intermediate i, purified after TCEP treatment, comprised one native inner disulfide bond between Cys18-Cys37. Acid-trapped intermediate i2 purified from the oxidative folding reaction displayed some degree of heterogeneity that hinders disulfide pairing analysis. As mentioned above, the coexistence of at least two species in this fraction was evident by judging the oxidative folding and reductive unfolding chromatographic profiles (see Fig. S5). Based on MALDI-TOF mass analysis, intermediate i1 comprises a non-native disulfide bond between Cys37-Cys47. According to the RP-HPLC oxidative folding profile (See Fig. 6), this non-native disulfide connectivity, that favors the exposure of hydrophobic residues, might be acting as a folding kinetic trap.

**Table 2:** Maldi-TOF analysis of Triap1 peptides after trypsin digestion and a previous sequential incubation of iodoacetamide (IAA) and vinylpyridine (VP)

Specie	Cysteine	Peptide sequence	TM (Da)	OM (Da)	Cys state
Native	Cys18	EYDQC∗IAA∗FNR	1131.431	1131.439	Oxidized
	Cys18	REYDQC∗IAA∗FNR	1287.532	1287.543	Oxidized
	Cys37	GDSSGDPC∗IAA∗TDLFK	1398.563	1398.578	Oxidized
	Cys47	YQQC∗IAA∗VQK	953.429	953.486	Oxidized
Int i	Cys8	MNSVGEAC∗VP∗TDMK	1390.522	1390.564	Reduced
	Cys18	EYDQC∗IAA∗FNR	1131.431	1131.433	Oxidized
	Cys18	REYDQC∗IAA∗FNR	1287.532	1287.536	Oxidized
	Cys37	GDSSGDPC∗IAA∗TDLFK	1398.563	1398.574	Oxidized
	Cys47	YQQC∗VP∗VQK	1001.429	1001.482	Reduced
Int i1	Cys8	MNSVGEAC∗VP∗TDMK	1390.522	1390.536	Reduced
	Cys18	EYDQC∗VP∗FNR	1179.431	1179.448	Reduced
	Cys18	REYDQC∗VP∗FNR	1335.532	1335.548	Reduced
	Cys37	GDSSGDPC∗IAA∗TDLFK	1398.563	1398.542	Oxidized
	Cys47	YQQC∗IAA∗VQK	953.429	953.47	Oxidized
Reduced	Cys8	MNSVGEAC∗VP∗TDMK	1390.522	1390.566	Reduced
	Cys18	EYDQC∗VP∗FNR	1179.431	1179.47	Reduced
	Cys18	REYDQC∗VP∗FNR	1335.532	1335.574	Reduced
	Cys37	GDSSGDPC∗VP∗TDLFK	1446.563	1446.604	Reduced
	Cys47	YQQC∗VP∗VQK	1001.429	1001.48	Reduced

Cysteines alkylated with VP correspond to free cysteines, and cysteines alkylated with IAA correspond to bridged cysteines when treated with trypsin.

OM, observable mass; TM, theoretical mass.

### Molten globule state of reduced TRIAP1 favors the accumulation of an off-pathway folding kinetic trap

To understand the role of the molten globule in guiding TRIAP1’s folding, we explore both the refolding and unfolding process under denaturing conditions. First, the conformational stability of TRIAP1 was assessed using the technique of disulfide scrambling. In this method, native disulfide-rich proteins are incubated with denaturants in the presence of a thiol initiator. As a consequence of their partial unfolding, they might reshuffle their native disulfide bonds and form disulfide-scrambled species (39, 40). Native TRIAP1 was incubated in the presence of 0.25 mM β-mercaptoethanol as a thiol initiator and increasing concentrations of urea, Gdm.HCl, or guanidine isothiocyanate (Gdm.SCN) at pH 8.4. The protein samples were allowed to reach equilibrium for 20 h, trapped by acidification, and analyzed by RP-HPLC (Fig. S7). As was observed previously for other small disulfide-rich proteins, denaturation was dependent on denaturant strength. Urea at 8.0 M was unable to promote Cys isomerization, while concentrations higher than 6.0 M Gdm.HCl or 4.0 M Gdm.SCN led to the accumulation of a major scrambled species exposing more hydrophobic residues as judged by RP-HPLC analysis (Fig. S7).

**Figure 7:**
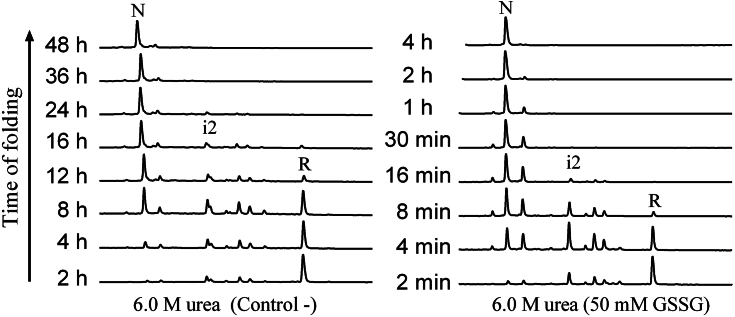
**Oxidative folding of TRIAP1 in the presence of 6.0 M urea.** The fully reduced/unfolded protein was allowed to refold in 0.1 M Tris.HCl (pH 8.4) in 6.0 M urea (*left panel*) or in the presence of 50 mM GSSG in 6.0 M urea (*right* panel). Intermediates were acid-trapped at the noted times and analyzed by RP-HPLC. The elution positions of native (N), reduced (R), and intermediate (i2) are indicated.

We next selected 6.0 M urea to perform the refolding experiment in the presence of denaturant, since TRIAP1 native state was still the predominant species and, accordingly, the most stable conformation in that condition. Besides, as shown in Figure 4*B*, 6.0 M urea impedes the population of the molten globule in reduced TRIAP1. Surprisingly, TRIAP1 refolding under denaturing conditions proceeded 2-times faster than in the absence of 6.0 M urea (Fig. 7*A*). The refolding reaction was almost completed after 12 h incubation, and the identity of the intermediates populated changed. Noteworthy, intermediate i1, which was the limiting species under nondenaturing conditions, was completely absent, indicating that conformational bias favors the accumulation of this disulfide species in the absence of urea, likely determined by the molten globule properties. In contrast, intermediate i2 accumulated to a greater extent than in nondenaturing conditions, together with some other minor species. When refolding was performed in 6.0 M urea containing 0.5 mM GSSG, the reaction was drastically accelerated and completed after 16 min (Fig. 7*B*). Intermediate i2 was the main species and a peak with a retention time close to the native two-disulfide peak accumulated during early stages of refolding and disappeared after 30 min incubation. This peak probably consists of the same scrambled species observed in disulfide scrambling experiments (Fig. S7), with compactness close to the native protein that isomerizes its disulfides to attain the energetically favorable two native disulfide species.

**Figure 8:**
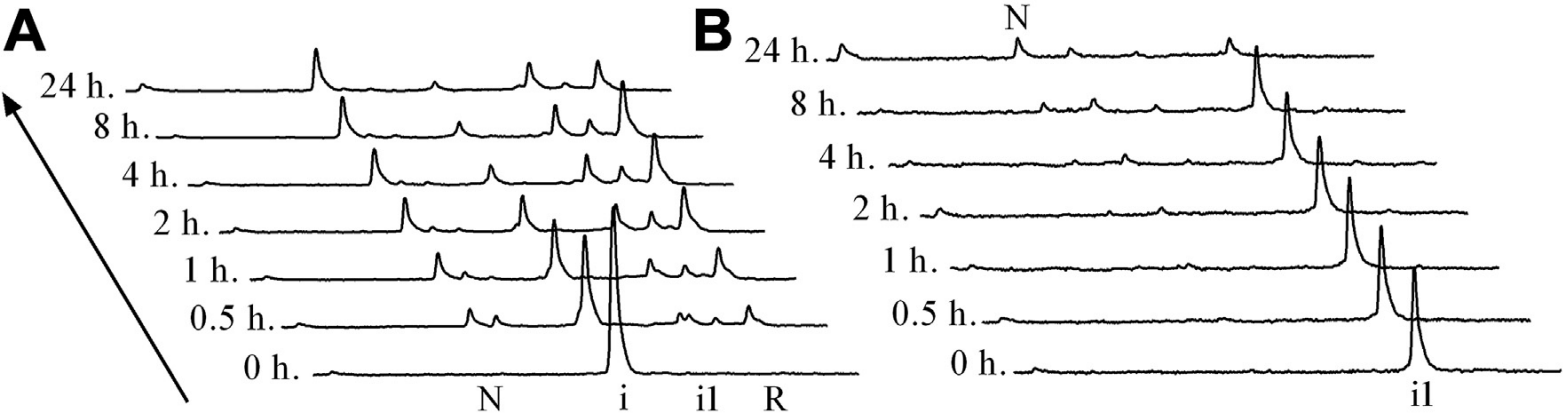
**Stop-Go refolding of TRIAP1 folding intermediates i1 and i.** TRIAP1 folding intermediates i1 and i were purified and allowed to resume their folding in 50 mM Tris.HCl (pH 8.4). Refolding intermediates were acid quenched at denoted time points and subjected to RP-HPLC analysis. Stop-Go refolding progression along time of i (*A*) and i1 (*B*) are shown.

To further assess the kinetic roles of TRIAP1 intermediates, we performed stop-go analysis. Purified intermediates, i and i1, were freeze-dried and allowed to resume their refolding at pH 8.4. Stop-Go RP-HPLC refolding profiles of i showed a rapid equilibrium with other refolding species, similar to those observed in TRIAP1 oxidative folding (Fig. 6, Control -), including the population of reduced species (Fig. 8). It seems likely that i promotes an exchange of disulfide bonds between TRIAP molecules to achieve the native structure. Conversely, after 4h of refolding, intermediate i1 neither showed progression of folding nor interconversion with other species, confirming that it acts as a kinetic trap.

The results described in this section suggest that (1) the molten globule of reduced TRIAP1 favors the formation of intermediate i1 (Cys37-Cys47 TRIAP1) and (2) intermediate i1 corresponds to an off-pathway intermediate acting as a kinetic trap in the folding reaction.

### MIA40 funnels the oxidative folding of TRIAP1, bypassing the kinetic trap

**Figure 9:**
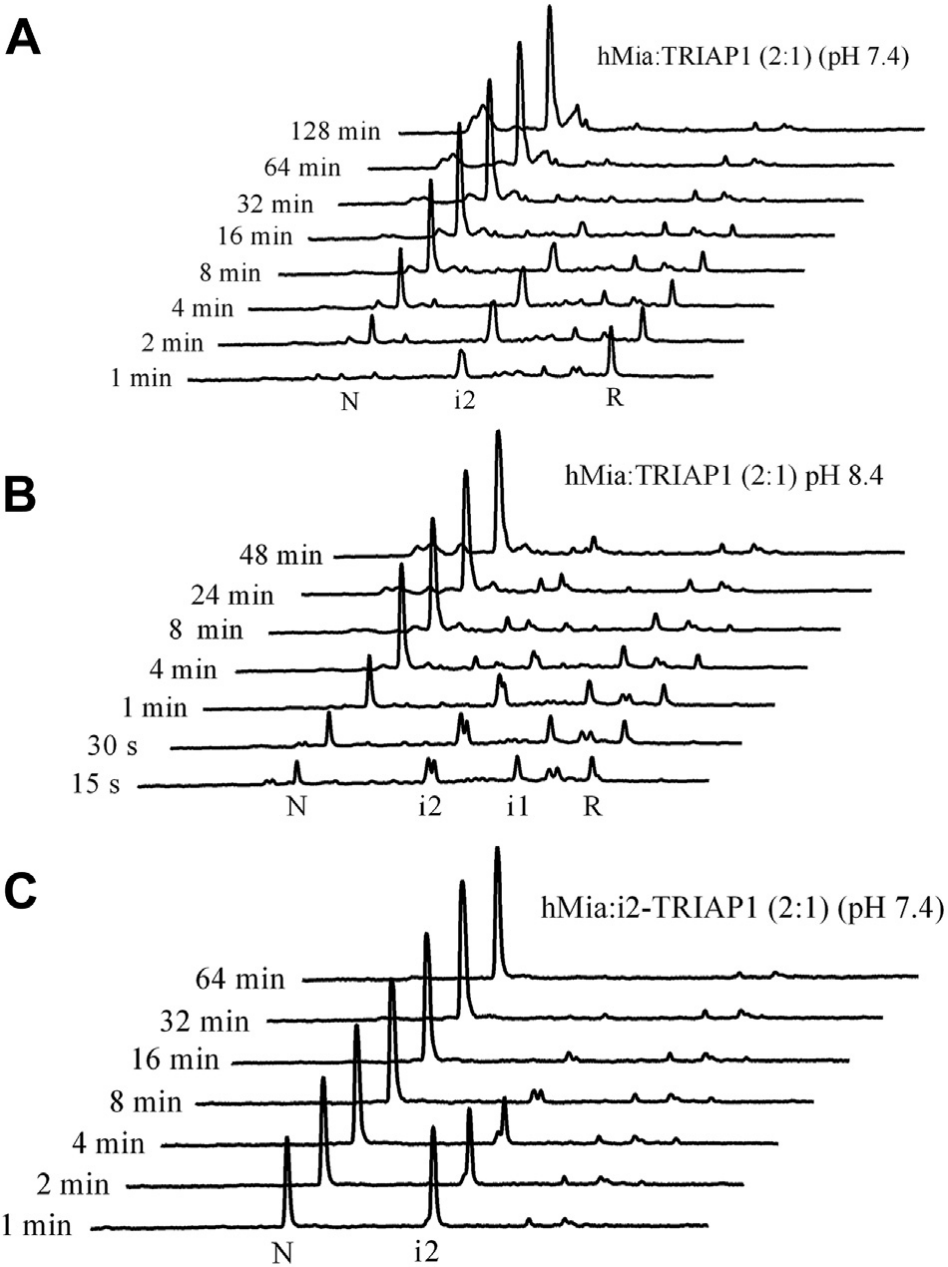
***In vitro* oxidative folding of reduced TRIAP1 or Cys18-Cys37 TRIAP1 catalyzed by hMia40.** GST-tagged hMia40 was added to reduced TRIAP1 (*Panels A* and *B*) or to intermediate i (Cys18-Cys37 TRIAP1) (*Panel C*), at a 2-fold molar excess in 50 mM Tris.HCl, 0.1 M NaCl pHs: 7.4 or 8.4 (indicated in each panel). Folding reactions were incubated at 20 °C, acid-quenched at the indicated time points, and subjected to RP-HPLC.

MIA40 catalyzes the formation of native disulfide bonds to newly imported polypeptides in the IMS (41). To gain insights into the chaperone-assisted oxidative folding process, we next analyzed the effect of MIA40 on TRIAP1 folding. Reduced TRIAP1 was allowed to refold in the presence of two-fold molar excess of GST-tagged MIA40 at two different pHs (pH 7.0 and pH 8.4). The samples were acid-trapped at selected time points and subjected to RP-HPLC to characterize the folding intermediates (Fig. 9). At pH 7.0, MIA40 catalyzed the rapid formation of mainly intermediate i2 (Cys18-Cys37 TRIAP1), which represented up to 50% of the species populating the reaction at 4 min of folding. The folding reaction was almost completed after 30 min incubation. At pH 8.4, chaperone-assisted oxidative folding progressed faster and other intermediates also accumulated, including i1. We hypothesize that MIA initially forms Cys18-Cys37, and the remaining free cysteines are highly reactive at pH 8.4, which in turn leads to a rapid isomerization, populating different intermediates. By stop-go experiments, we have shown above that intermediate i is unable to form the second disulfide bond by air oxidation. Alternatively, the highly reactive free Cys8 or Cys47 residues of Cys18-Cys37 TRIAP1 rapidly isomerize to give rise to several intermediates that ultimately lead to native TRIAP1. In this context, we asked whether MIA40 was able to catalyze the formation of the outer native disulfide bond. Purified intermediate i (Cys18-Cys37 TRIAP1) was freeze-dried and resuspended in a two-fold molar excess of MIA to characterize the progression of the reaction over time. As shown in Figure 9*C*, MIA40 was able to rapidly introduce the second outer disulfide bond to give rise to the native protein.

Finally, to further gain insight into the mechanism and specificity of chaperone-assisted oxidative folding, TRIAP1 refolding was performed in the presence of the yeast ortholog Mia40 (yMia40). The RP-HPLC chromatographic profiles of the folding reaction were similar to those observed for human MIA40, confirming that the hydrophobic recognition of substrates, and the chaperone-assisted oxidative folding pathway are common features among ortholog chaperones (Fig. S8). Moreover, yMia40 was also able to catalyze the formation of the outer disulfide bond (Fig. S9).

To rule out any artificial impact of GST disulfide bonds on the TRIAP1 oxidative folding pathway, reduced TRIAP1 was co-incubated with GST. No significant differences in folding intermediates or kinetics were observed compared to O_2_-catalyzed reactions (Fig. S10).

## Discussion

Since the seminal work of C. Anfinsen with RNase, the study of disulfide-rich proteins has provided data to advance our knowledge on the molecular principles that govern protein folding (1). These proteins have been useful models to overcome methodologic limitations in protein folding studies, such as the inaccessibility to unfolded states in native buffering conditions or the transient nature of folding intermediates (42). The emergence of new concepts like protein disorder, conditional folding, and functional moonlighting suggested that apart from the well-established role of disulfide bonding in defining and stabilizing the native structure, they may impact physiologic processes acting as inducible and reversible post translational modifications (43, 44). Several elegant works point towards this direction, with disulfide-rich proteins shifting between active and inactive conformations in a redox-controled manner (45, 46).

The IMS's disulfide-rich proteins illustrate this mechanism well since they populate two different and equally relevant conformations in the cell (4, 21): a cytosolic reduced import-competent state and a mitochondrial-oxidized folded and functional state. For these proteins, synthesized in the cytosol, oxidation is compartment-specific and promoted by the specific binding and catalysis of MIA40. For a long time, it has been accepted that, when reduced, these proteins were mostly disordered, which would facilitate a free-energy import across the TOM. Our data uncover distinctive characteristics of TRIAP1 that distinguish it from typical CX9C MIA40 substrates, since it exhibits an unusual hydrophobic load and high alpha-helix propensity, when compared with other MIA40 substrates. Our results also show that reduced TRIAP1 collapses into a compact conformation with a significant content of secondary structure and hydrophobic patches exposed to solvent, instead of a mostly extended disordered conformation. This metastable conformation is also dynamic, as shown by NMR. Altogether, these properties indicate that reduced TRIAP1 resembles a collapsed early-stage intermediate, classically referred to as molten globule. Far from being a marginal structural arrangement, TRIAP1 molten globule state corresponds to a thermodynamically metastable conformation that exhibits folding cooperativity.

**Figure 10:**
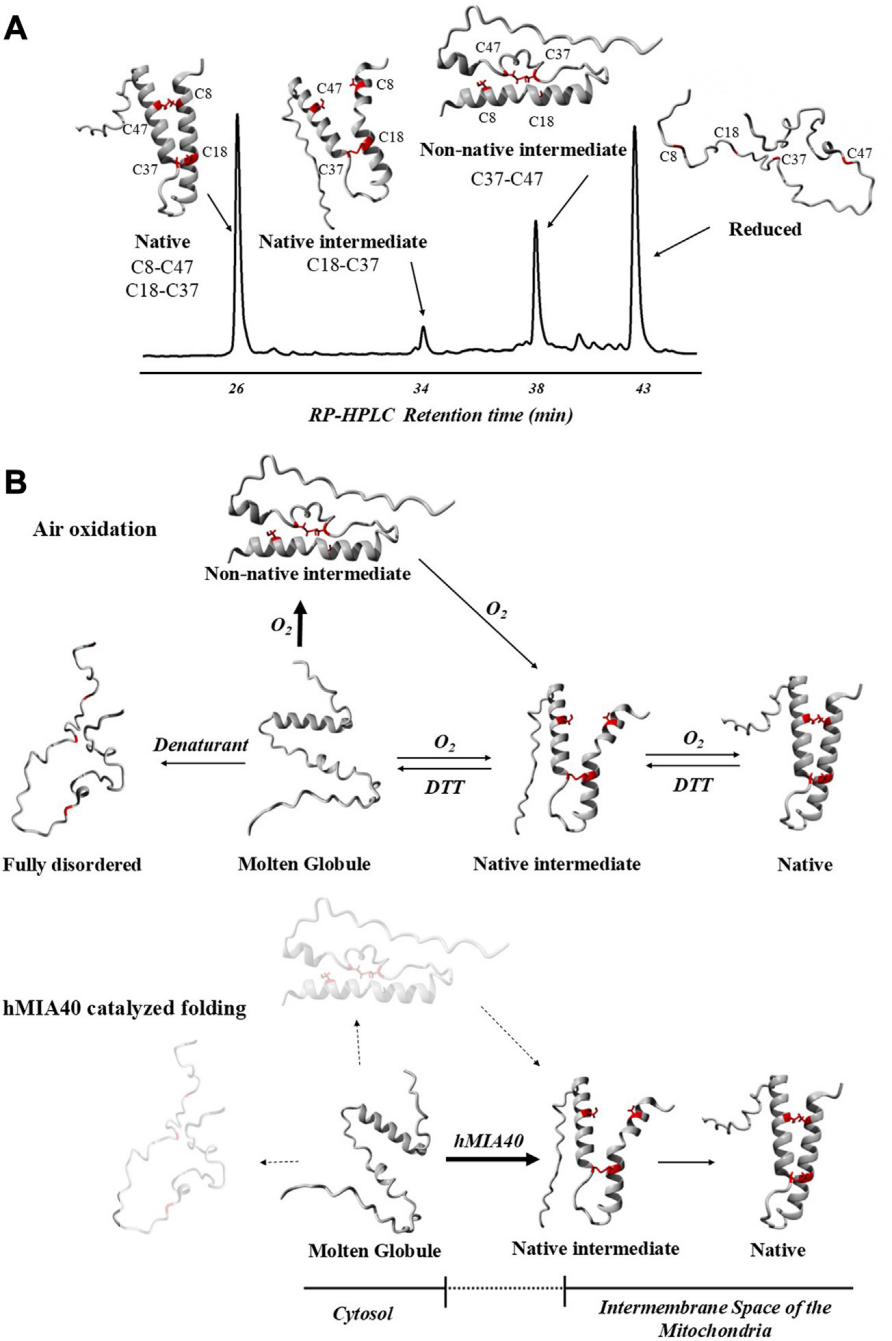
**Schematic representation of the O2-catalyzed and chaperone-assisted disulfide oxidative folding pathways of TRIAP1.***A*, TRIAP1 redox species correlation with RP-HPLC retention times. The TRIAP1 species are abstract representations based on pdb:6I3Y. Cysteine residues are indicated and labeled in *red*. *B*, schematic folding pathway of TRIAP1 catalyzed by O_2_ (*upper panel*) and by hMIA40 (*bottom panel*). The proposed cellular localization of the molten globule and the folded versions of TRIAP1 are indicated in the hMIA40 panel.

As we previously reported for other hMIA40 substrates (27, 28, 47), we reveal that TRIAP1 can fold into its native state by simple air-oxidation, although in an extremely slow and inefficient reaction. Contrary to COX17, whose folding proceeds through native-like intermediates (27), we demonstrate that the population of the TRIAP1 molten globule frustrates its folding landscape, guiding the accumulation of a non-native intermediate (i1) mislead by Cys37-Cys47 disulfide. By RP-HPLC analysis, we have shown that i1 elutes close to the reduced species, suggesting a high amount of exposed hydrophobic surfaces. Stop-go experiments and refolding in the presence of denaturant revealed that i1 acts as an off-pathway folding kinetic trap. Free cysteines of i1, Cys8 and Cys18, are likely buried and not accessible to react, but when this conformation is perturbed by denaturing conditions in 6.0 M urea or TRIAP1 departs from a fully disordered conformation in the presence of denaturant, intermediate i1 no longer accumulates and consequently, folding proceeds faster (Fig. 10*A*).

A similar effect was observed in the presences of hMIA40, which acts as a catalyst and bypasses the formation of the non-native disulfide. Using as substrate for the fully reduced TRIAP1, we have shown that hMIA40 initially drives the formation of the inner Cys18-Cys37 disulfide, assessed by the accumulation of intermediate i2 in RP-HPLC profiles. Most interestingly, when Cys18-Cys37 TRIAP1 was used as hMIA40 substrate, the chaperone rapidly drives the formation of the outer Cys8-Cys47 disulfide (Fig. 10*B*). In line with our results, using purified MIA40 and COX19, Bien *et al.* showed that hMIA could sequentially introduce both disulfide bonds in its substrate. Initially, a long-lived disulfide-linked hMIA40–COX19 complex is formed, which is rapidly resolved by GSH (48). One limitation of our RP-HPLC–based oxidative folding analysis is the inability to resolve GST–hMIA40–TRIAP1 mixed complexes, as GST-hMIA40 remains strongly bound to the C4 resin and only elutes at high acetonitrile concentrations.

Whether by specific or nonspecific interactions, hMIA40 likely positions and forms a mixed disulfide between the second Cys of the catalytic CPC site and one of the free cysteines of Cys18-Cys37 TRIAP1. This is supported by findings showing hMIA40 plasticity in substrate recognition that allows the formation of disulfide bonds with various cysteine residues in its substrates (24). It is important to note that when purified i intermediate Cys18-Cys37 TRIAP1 was allowed to refold *in vitro* by air-oxidation, it isomerizes to give rise to the conjunct of species populating the oxidative folding pathway. Indeed, the hMIA40-catalyzed reaction at pH 8.4 also revealed an accumulation of minor species, such as intermediate i1, and in addition, an intermediate that broadened the elution peak of i. We speculate that once hMIA40 drives the formation of the inner disulfide, one of the free cysteines, namely Cys8 or Cys47, is highly reactive at pH 8.4 and rapidly isomerizes to other species. The high reactivity of these cysteines was also evidenced in reductive unfolding experiments at distinct pHs. Upon TCEP treatment at pH 5.0, while the outer disulfide bond was easily accessible for reduction, the inner disulfide was not. This leads to a large accumulation of i (Cys18-Cys37 TRIAP1), in a condition where both free cysteines are likely protonated as thiols. However, at pH 8.4, once the outer disulfide is reduced, thiolates of free Cys residues might react rapidly with the inner disulfide bond, which in turn exposes the second disulfide to the reducing agent, resulting in a very low accumulation of i. We suggest that the tendency of TRIAP1 to collapse into a molten globule-like arrangement is permanently constraining its folding pathway.

Beyond the unreported role of the molten globule state in defining the folding pathway of a MIA40 substrate, it is important to reframe to which extent this compacted state has an actual impact on the biological context. Interestingly, MIA40 catalyzes the oxidation of many small disulfide-rich proteins bearing CX_3_C and CX_9_C twin motifs. All of them have been classically considered as mostly disordered polypeptides in their reduced state, to be easily translocated across the TOM. Precisely, it is suggested that the mixed species MIA40-substrate acts as a pulling mechanical force towards the IMS (23, 41). In this context, we hypothesized that these families of proteins undergo folding upon binding as they are recognized by the hydrophobic cleft of MIA40 in their disordered state. However, in the case of TRIAP1, MIA40 must recognize a folded metastable state, a behavior that has been reported for noncanonical substrates of MIA40, primarily larger proteins that do not bear MIA40 recognition sequences (49, 50). Our results align with experimental evidence that highlights MIA40 as a versatile oxidoreductase that accommodates multiple topologies and promotes the disulfide bridging of several noncanonic substrates (3, 41). This remarkable adaptability towards different substrates confers MIA40 a pivotal role within the protein import system to the IMS. In this context, given the nature of TRIAP1, it agglutinates all the characteristics to serve as a model system to understand the functioning of the disulfide relay system in these atypical cases.

The inherent question is why does TRIAP1 maintain sequential features that deviate from those of canonical MIA40 substrates? Proteins involved in both obligatory and transient protein complexes exhibit expose hydrophobicity on their surfaces when in isolation, which becomes hidden at the interface once the complex is formed (51, 52, 53). The hydrophobicity of TRIAP1 is evolutionary conserved and responds to a functional requirement. Native TRIAP1 needs an exposed hydrophobic cleft to stabilize its partners in the IMS—the members of the PRELI-like family—thus explaining why hydrophobicity could not be purged out through natural selection. We have demonstrated that the amino acid composition of small disulfide-rich proteins, and particularly of MIA40 substrates, resemble that of IDPs (29). In the absence of a protective tertiary structure, hydrophobic residues in IDPs remain exposed to the solvent and ready for non-native intermolecular interactions. Accordingly, IDPs display sequences of low hydrophobicity, which enables them to minimize the risk of aggregation (54). For most MIA40 substrates, this inherent low hydrophobicity allows them to transit in a reduced and disordered state from the cytosol to the mitochondria, circumventing undesired aggregation reactions. They do not rely on a hydrophobic core for folding, as the driving force behind this process is the oxidation of a native disulfide after binding to MIA40 in the IMS (41). In the case of TRIAP1, functional constraints cause it to deviate from these generic features, forcing the collapse of the polypeptide chain into a partially folded hydrophobic molten globule. Importantly, it has been demonstrated that molten globule-like states are particularly prone to aggregation.

Cellular protective systems, such as the quality control machinery, are aimed to prevent aggregation, refold misfolded species, or promote their degradation. Particularly, the chaperone HSP70 recognizes partially folded species by binding to abnormally exposed hydrophobic regions (55). In this context, is important to note that the cytosolic function of TRIAP1 as an apoptotic repressor by p53-dependent expression stems from its interaction with HSP70, impeding cytochrome c binding to Apaf-1 and the activation of caspase-9, ultimately inhibiting apoptosis and allowing for DNA damage repair (18). Our findings support the notion that upon translation in the reducing environment of the cytoplasm, TRIAP1 would adopt a molten globule state, which might be subsequently recognized by the HSP70 machinery (56). This interaction likely connects TRIAP1 and the p53-dependent cell survival pathway.

Apoptosis evasion is one of the hallmarks of cancer (57). TRIAP1 is thought to be transported passively to the IMS. Therefore, an increase in protein synthesis, without an associated increase in protein efflux to the mitochondria, inevitably would lead to the accumulation of TRIAP1 in its molten globule state within the cytosol. This accumulation might hijack HSP70 and subsequently inhibit apoptosome formation. This can be the underlying mechanism explaining why TRIAP1 expression is upregulated in a variety of malignancies (58) and associated with poor patient survival (59, 60). Furthermore, it provides a possible explanation for the correlation between elevated TRIAP1 levels and drug resistance (19), as cancer cells often develop resistance to therapeutic interventions through apoptosis evasion. However, additional experimental evidence is required to establish the presence of a functional cytoplasmic-reduced molten globule state of TRIAP1 in the context of both normal physiological conditions and cancer-associated contexts.

Overall, this study shed light into the structural duality of TRIAP1, a feature that might explain its compartment-specific function. A redox-controled structural transition resolves a folding kinetic trap by the assistance of the mitochondrial chaperone and oxidoreductase MIA40. TRIAP1 stands as a model to study the conformational properties of the often-elusive molten globule state. Additionally, this protein is an amenable model to understand the import of MIA40 noncanonical substrates into the IMS. Although further experimental validation is required, the identification of this early folding intermediate as an apoptosis-repressing species would represent the first description of a functional molten globule conformation.

## Experimental procedures

### Proteins expression and purification

Human TRIAP1 complementary DNA was cloned into pET28b(+) vector as C-terminal 6xHis-tagged fusion protein and the resulting plasmid was sequenced and transformed into *Escherichia coli* Origam2 cells. Cells were grown in LB broth at 37 °C and protein expression was induced at A_600nm_ = 0.8 with 1 mM IPTG and the culture was incubated overnight (ON) at 20 °C. Cells were harvested by centrifugation, the pellet was resuspended in 0.1 M Tris.HCl pH 8.0, 0.5 M NaCl, and lysed by sonication. The supernatant was purified using a nickel-affinity chromatography followed by a size-exclusion chromatography in a HiLoad 26/60 Superdex 75 prep-grade column (GE healthcare) pre-equilibrated in 20 mM Tris.HCl pH 8.0 and 0.1 M NaCl.

For ^15^N-labeled TRIAP1 production, TRIAP1 expression vector was transformed into *E. Coli* BL21 cells and grown in M9 minimal media, supplemented with 4 g of ^15^NH_4_Cl per liter. Protein expression was induced adding 1 mM IPTG at A_600nm_ = 0.8. Following induction, the culture was incubated ON at 20 °C. ^15^N-labeled TRIAP1 was purified as previously described for the nonlabeled protein.

The purity of the isolated TRIAP1 protein was determined by Tris-Tricine/SDS-PAGE, RP-HPLC, and MALDI-TOF mass spectrometry (MS). The concentration of purified TRIAP1 was determined by measuring the absorbance at 280 nm using a molar extinction coefficient of 8730 M^−1^ cm^−1^. The catalytic Mia40 C-terminal domain was purified as an N-terminal GST fusion protein from *E. coli* cells as was previously described (28).

For human Mia protein expression and purification, hMia sequence (Uniprot ID: Q8N4Q1) was cloned into pGEX-5X, resulting in a fusion protein with an N terminal GST tag, the hMIA sequence, and a C-terminal 6X His tag. Cysteine four of hIMA was mutated to alanine to avoid disulfide isomerization with the CPC active site BL21. DE3 pLys cells were transformed with the expression vector, grown in LB broth at 37 °C. Protein expression was induced at A600 nm = 0.6 with 1 mM IPTG and incubated ON at 20 °C. Cell pellet was resuspended in 0.1 M Tris.HCl pH 8.0, 0.5 M NaCl and lysed by sonication. The supernatant was purified using a nickel-affinity chromatography followed by a size-exclusion chromatography in a HiLoad 26/60 superdex 75 prep-grade column pre-equilibrated in 20 mM Tris.HCl pH 8.0 and 0.1 M NaCl.

### Oxidative folding and reductive unfolding experiments

Purified native TRIAP1 (1 mg/ml = 101 μM) was reduced and unfolded by incubation in 0.1 M Tris.HCl (pH 8.4) containing 200 mM DTT and 6.0 M Gdm.HCl for 3 h at RT. To initiate refolding, the fully reduced/unfolded protein samples were loaded onto a Hi-Trap desalting column (GE Healthcare) connected to an AKTA purifier system, previously equilibrated in 0.1 M Tris.HCl (pH 8.4). The protein was eluted in 1.2 ml of equilibration buffer (protein concentration ∼ 0.5 mg/ml) and incubated at RT in the absence (Control -) and in the presence of redox agents: 0.25 mM 2-mercaptoethanol (Control +), 0.5 mM GSSG, or 0.5 mM/1.0 mM GSSG/GSH. Refolding experiments were also performed in 6.0 M urea in the absence or presence of redox reagents. The refolding reaction was monitored by removing aliquots of the sample at selected time-points and quenching them with 1% aqueous trifluoroacetic acid (TFA). Acid-trapped intermediates were subsequently analyzed by RP-HPLC using a linear 25 to 40% gradient of acetonitrile with 0.1% TFA over 50 min in a 4.6-mm C4 column (Phenomenex) at a flow rate of 0.75 ml/min. Reductive unfolding experiments were performed by incubating native TRIAP1 (15 μM) at RT upon the addition of: 1 mM DTT in 0.1 M Tris.HCl pH 8.4, 5 mM DTT in 0.1 M sodium phosphate pH 7.4, and 32 mM TCEP in 0.1 M sodium acetate pH 5.0. To monitor the progression of disulfide reduction, time course aliquots of the samples were trapped with 1% TFA acid and analyzed by RP-HPLC in a C4 column in the same conditions described above.

### Far-UV CD and fluorescence spectroscopy

The conformational changes along oxidative folding or TRIAP1 disulfide reduction were followed by far-UV CD and tryptophan fluorescence spectroscopy. To monitor the oxidative folding reaction, the sample was prepared by dissolving the fully-reduced/unfolded protein to a final concentration of 15 μM in 20 mM Tris–HCl (pH 8.4). CD spectra were collected at 20 min intervals in a spectropolarimeter (Jasco J-710) at 25 °C using an optical path length of 1 mm. Samples for fluorescence spectroscopy were measured in the same buffer on a fluorescence spectrophotometer (Jasco FP-8500) at 25 °C. The spectra were measured every 20 min in the 310 to 450 nm interval using a 294-nm excitation wavelength (5 nm excitation and emission slits, 0.1 s averaging time). For disulfide reduction, native TRIAP1 samples were prepared at 15 μM in 50 mM Tris.HCl pH 8.4, 0.1 M sodium phosphate (pH 7.4), or 0.1 M sodium acetate (pH 5.0). After measuring the native protein spectrum, the reducing agent was added, without diluting the samples, to a final concentration of 5.0 mM DTT (pH 8.4), 1.0 mM DTT (pH 7.4), and 32 mM TCEP (pH 5.0). Tryptophan emission spectra were measured at various time points in the same conditions described for oxidative folding. The secondary structure changes were followed at 222 nm, upon the addition of the reducing agent.

For urea equilibrium denaturation measurements, a TRIAP1 stock solution was first incubated for 2 h at RT in 50 mM Tris.HCl containing 10 mM DTT. Then, the samples were diluted in 50 mM Tris.HCl pH 8.0 at 20 μM TRIAP1 and 1.0 mM DTT and increasing urea concentrations in the absence or presence of 0.5 and 1.0 M TMAO. Samples were incubated for 16 h at 5 °C prior measurements. Tryptophan fluorescence emission spectra were recorded between 310 and 450 nm with excitation wavelength of 294 nm. CD spectra were recorded between 210 and 260 nm at a rate of 100 nm/min, a response of 2 s, and a bandwidth of 2 nm. Spectroscopic data were analyzed after subtracting the buffer background composition. Measurements were taken at 5 °C.

### NMR measurements

Solution NMR experiments were performed on [U- ^15^N] isotopically enriched samples of TRIAP1 at 0.3 mM in 20 mM sodium phosphate buffer, pH 7.0, containing 10% of D_2_O. To record the spectra of reduced TRIAP1, 0.1 M DTT was added and incubated ON at room temperature before measurements. Complete denaturation of reduced TRIAP1 was obtained by adding 6.0 M urea and 0.1 M DTT followed by ON incubation at RT. All solution NMR spectra were recorded at 298 K either on a Bruker AVANCE NEO NMR spectrometer, operating at 700 MHz, ^1^H Larmor frequency, (16.4 T), equipped with a triple resonance cryo-probe or an 800 MHz Agilent DD2 spectrometer equipped with a conventional triple resonance probe. The data were processed with nmrPipe (61).

### Urea equilibrium denaturation fit to two-state model

To estimate the thermodynamic parameters for the urea denaturation transitions, denaturation data of reduced TRIAP1 in the presence of increasing concentrations of TMAO was fit to a two-state denaturation model as was previously described (62, 63).

For monitoring transitions, we used the raw ellipticity at 222 nm and the CSM of tryptophan emission fluorescence spectrum that is calculated by the following equation:

(1) $$\text{CSM}\;\left(\text{cm}-1\right)=\frac{\sum \left(\sigma i*\text{Fi}\right)\;}{\sum \text{Fi}}$$

Where Fi is the fluorescence emission at wave number σi and the summation is carried out over the range of measured values of F.

Thermodynamic parameters were determined by a nonlinear curve fitting to the following equation:

(2) $$y=\frac{\left[\text{Sn}\;+\;\text{Su}\;\exp\left(\frac{{-\Delta G}_{H20}\;+\;\text{mx}}{\text{RT}}\right)\right]}{1\;+\;\exp\;\left(\frac{{-\Delta G}_{H20}\;+\;\text{mx}}{\text{RT}}\right)}$$

where y is the observed fluorescence or CD signal; Sn and Su represent the spectroscopic signals of folded and unfolded proteins, respectively; ΔG and m represent the free-energy change and slope of the transition, respectively; x is the denaturant concentration; T is the temperature (in K), and R is the universal gas constant (1.987 kcal/mol). TMAO stabilization of molten globes was shown to take place without changing the baselines of the unfolding curves or the m-value of the process (36, 38). Thus, urea denaturation curves at 0, 0.5, and 1.0 M TMAO were analyzed individually assuming that the pre- and post-transition baselines and the m-values are the same at all TMAO concentrations.

### Disulfide scrambling

The native TRIAP1 protein (15 μM) was dissolved in 0.1 M Tris.HCl pH (8.4) containing 0.25 mM 2-mercaptoethanol and increasing concentrations of denaturants: urea (0–8 M), Gdm.HCl (0–7 M), or Gdm.SCN (0–5 M). The samples were incubated for 20 h at RT to reach equilibrium and quenched with 1% TFA acid for further RP-HPLC analysis, as detailed in oxidative folding. The disulfide scrambling experiments were performed in duplicates at RT.

### Disulfide-bond content and disulfide pairing analysis of the major intermediates of oxidative folding and reductive unfolding of TRIAP1

The acid-trapped intermediates were purified by RP-HPLC and freeze-dried. The homogeneity of each species was controlled by RP-HPLC. The free cysteines of the purified intermediates (20–40 μg) were blocked by derivatization with 0.1 M 2-Vinylpyridine in Tris.HCl (pH 8.4) at RT for 45 min in the dark. An aliquot of the samples was analyzed by MALDI-TOF-MS, to characterize the number of disulfide bonds of the folding intermediates. The derivatized samples were then freed from reagents using Zip-Tip C4 pipette tip (Millipore), evaporated, and the disulfide bonds were reduced by incubation for 1 h at RT of the dried samples resuspended in 10 μl of 0.1 M Tris.HCl (pH 8.4) and 10 mM DTT. The free cysteines, corresponding to those initially involved in disulfide bridges, were then subjected to a second alkylation reaction by adding 10 μl of 0.05 M IAA in 0.1 M Tris.HCl (pH 8.4). Then, the double-derivatized samples were purified by Zip-Tip C4 and air-dried. The dried samples were resuspended in 10 μl of ammonium bicarbonate (pH 8.8) containing 0.15 μg of MS grade trypsin (Trypsin Gold, Promega) and were incubated for 16 h at 37 °C. The resulting derivatized digestion peptides were analyzed by MALDI-TOF-MS.

### Stop/Go folding and Mia40-catalyzed reaction of Cys18-Cys37 TRIAP1 intermediate

Intermediate i (Cys18-Cys37 TRIAP1) was isolated as follows: TRIAP1 (1 mg/ml) was reduced by adding to the reaction 16 mM TCEP in 0.1 M sodium acetate (pH 5.0). After 30 min incubation at RT, the sample was acid quenched and subjected to RP-HPLC. The intermediate was isolated, lyophilized, and quantified by RP-HPLC using a standard calibration curve of TRIAP1. Lyophilized intermediate was allowed to reinitiate folding at 15 μM in 0.1 M Tris.HCl (pH 8.4), 0.1 M NaCl at RT (Control -). The Mia40-catalyzed reaction was performed by adding 30 μM of GST-Mia40 fusion protein to 15 μM of purified intermediate i in 0.1 M Tris.HCl (pH 8.4), 0.1 M NaCl at RT. Time-course aliquots of the folding reactions were trapped with 2% TFA acid and analyzed by RP-HPLC in a C4 column using the same gradient described for oxidative folding.

### MALDI-TOF analyses

MALDI-TOF mass spectra were recorded on an UltrafleXtreme mass spectrometer (Bruker Daltonics) and the samples were spotted onto a MALDI-TOF-MS ground steel plate using the dried-droplet method. For peptide analyses, the samples were mixed with α-cyano-4-hydroxycinnamic acid and acquired in the positive ion reflectron mode, with ion acceleration set to 25 kV. All mass spectra were externally calibrated using a standard peptide mixture (Bruker Daltonics). For protein analyses, the samples were mixed with sinapic acid and acquired in the positive ion lineal mode, with ion acceleration set to 25 kV. All mass spectra were externally calibrated using a standard protein mixture ranging from 4 kDa to 20 kDa (Bruker Daltonics).

## Data availability

The data supporting the findings of this study are included in the manuscript and its supplementary material section. Any additional information or data are available upon request. Contact information: e-mail: salvador.ventura@uab.es.

## Supporting information

This article contains supporting information.

## Conflicts of interests

S. A. E. is a member of the research career of the National Scientific and Technical Research Council (CONICET), Argentina. All authors declare that they have no conflicts of interests with the contents of this article.
