# Supplementary material for: MIA40 circumvents the folding constraints imposed by TRIAP1 function
Source: J Biol Chem. 2025 Feb 3;301(3):108268. doi: 10.1016/j.jbc.2025.108268 (PMC11930124; doi:10.1016/j.jbc.2025.108268)
Supplement: Supplementary data [file mmc1.pdf]

**Supplementary Materials**

# MIA40 circumvents the folding constraints imposed by TRIAP1 function.

Jordi Pujols, Marc Fornt, Marcos Gil-García, Andrea Bartolomé-Nafría, Francesc Canals, Linda Cerofolini, Kaare Teilum, Lucia Banci, Sebastián A. Esperante ✉, Salvador Ventura ✉.

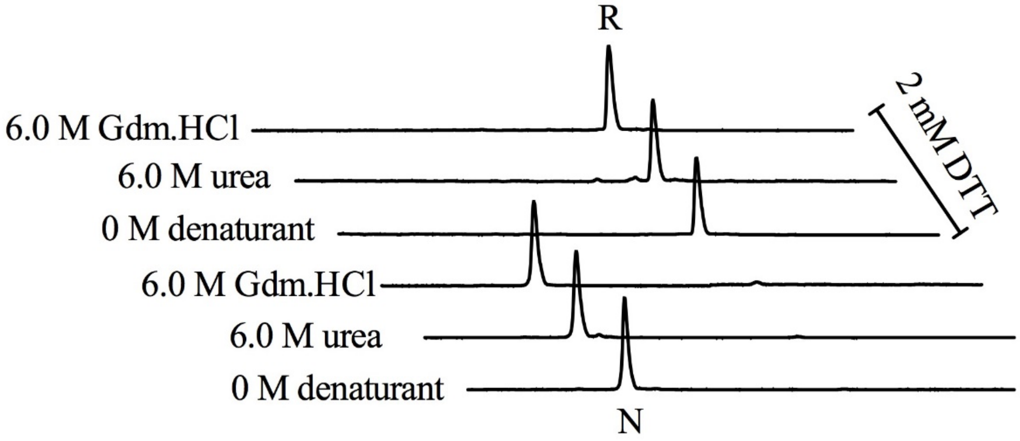


**Figure S1. RP-HPLC chromatographic profiles of *native, fully reduced and fully reduced/unfolded TRIAP1.*** 15 μM of TRIAP1 samples were incubated at RT for 20 h in: 50 mM sodium phosphate pH 7.4 containing: 0 M denaturant; 6.0 M urea or 6.0 M Gdm.HCl in the absence or presence of 2 mM DTT. The samples were acid-quenched and subjected to RP-HPLC analysis. The incubation condition of each sample is shown. The native (N) and the fully reduced (R) peaks are indicated.

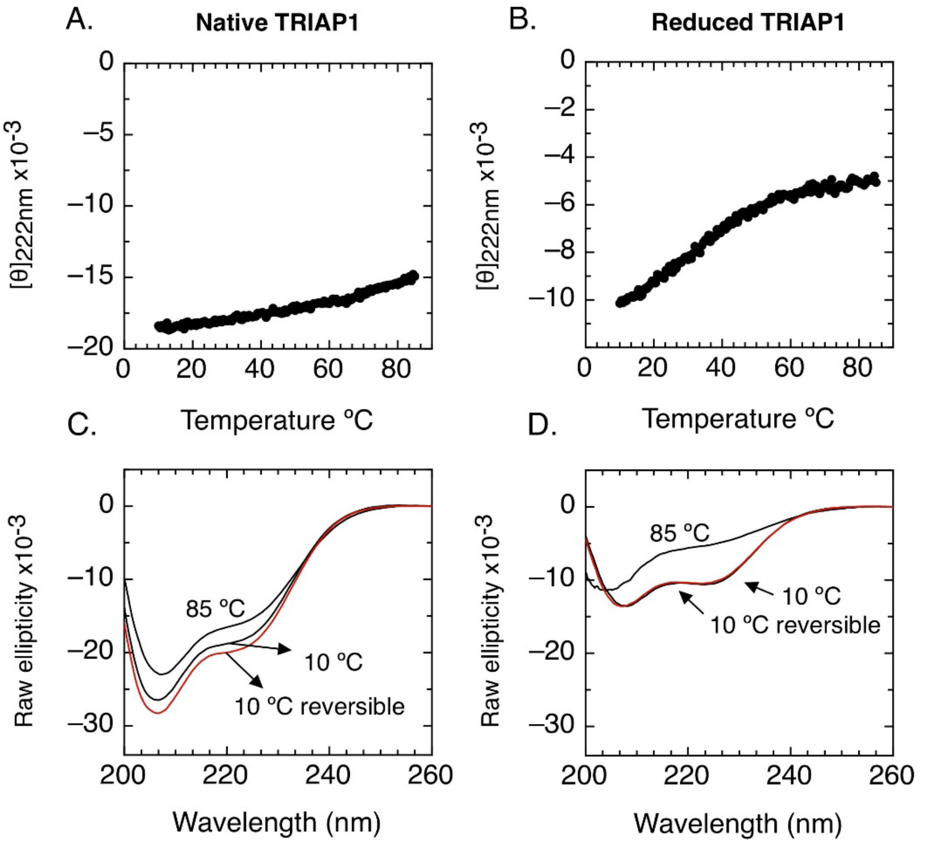


**Figure S2. Thermal denaturation of native and fully reduced TRIAP1.** The native (A) and fully reduced (B) TRIAP1 samples at 15 µM were dissolved in 50 mM sodium phosphate (pH 7.4) and the temperature gradually increased from 10 ºC to 85 ºC, at a heating rate of 1.0 ºC $min^{-1}$. The changes in the ellipticity at 222 nm were monitored every 0.5 ºC. (C, D) Spectra of the samples were taken at the initial and final temperatures (10 and 85 ºC, respectively). The samples were equilibrated at 10 ºC after the thermal denaturation to confirm the reversibility of the reaction.

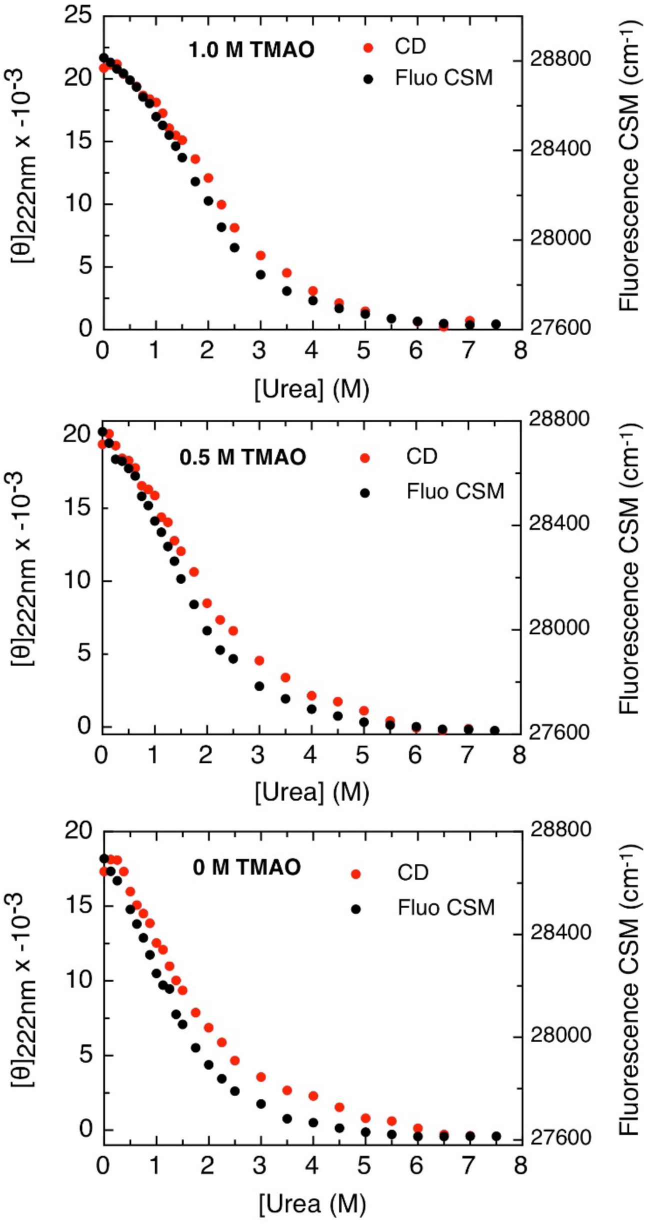


**Figure S3 Uncoupling of fluorescence and far-UV CD signals of reduced TRIAP1 molten globule equilibrium urea denaturation.** Urea equilibrium denaturation curves of 20 µM TRIAP1 were performed in the absence (Panel C) or presence of 0.5 and 1.0 M TMAO (Panels B and A, respectively) at 5 $^{o}$C. Denaturation was followed by Far-UV CD at 222 nm (left axis, red dots) and by tryptophan fluorescence CSM (right axis, black dots). Experiments were performed twice, with similar results, a representative experiment is shown.

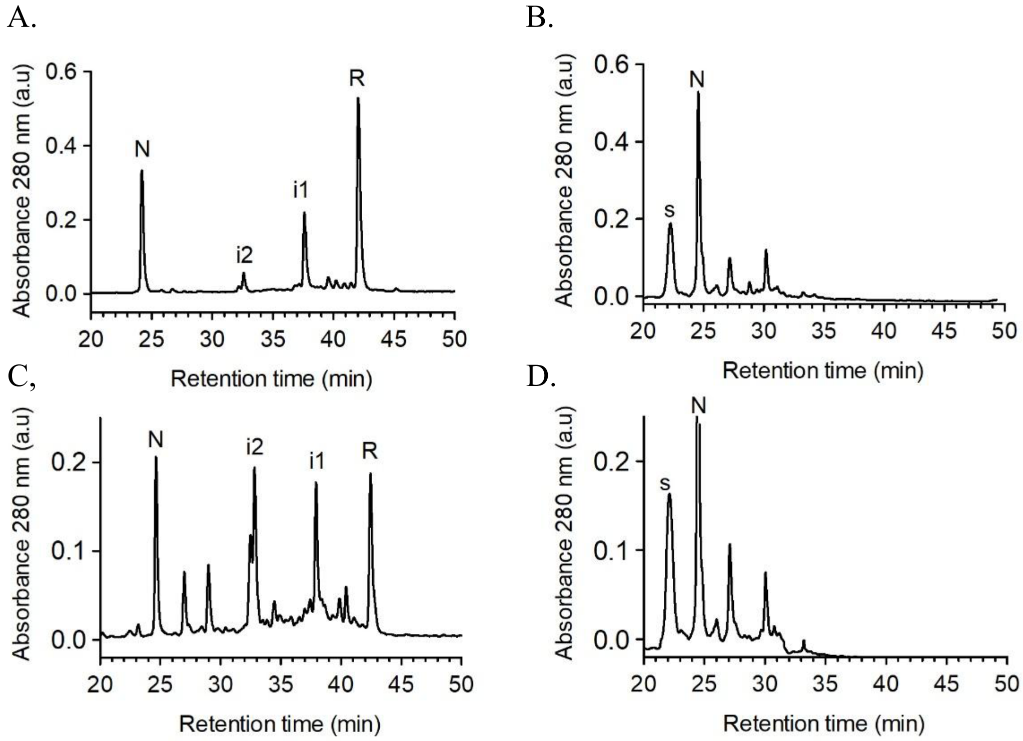


**Figure S4.** ***Comparison of RP-HPLC chromatographic profiles of TRIAP1 oxidative folding intermediates.*** RP-HPLC chromatographic profiles of selected acid-quenched TRIAP1 refolding reactions at different time points and incubating conditions: (A) 8 h, control; (B) 4 min, 1 mM GSSG; (C) 1 h and (D) 2 h, in the presence of 0.5 mM GSH and 1 mM GSSG. The native (N), reduced (R), intermediates (i1 and i2) and scramble (S) peaks are indicated. The heterogeneity of peak i2 is clearly observed in panel C. The accumulation of scramble species along incubation is clearly observed by comparing panels B and D.

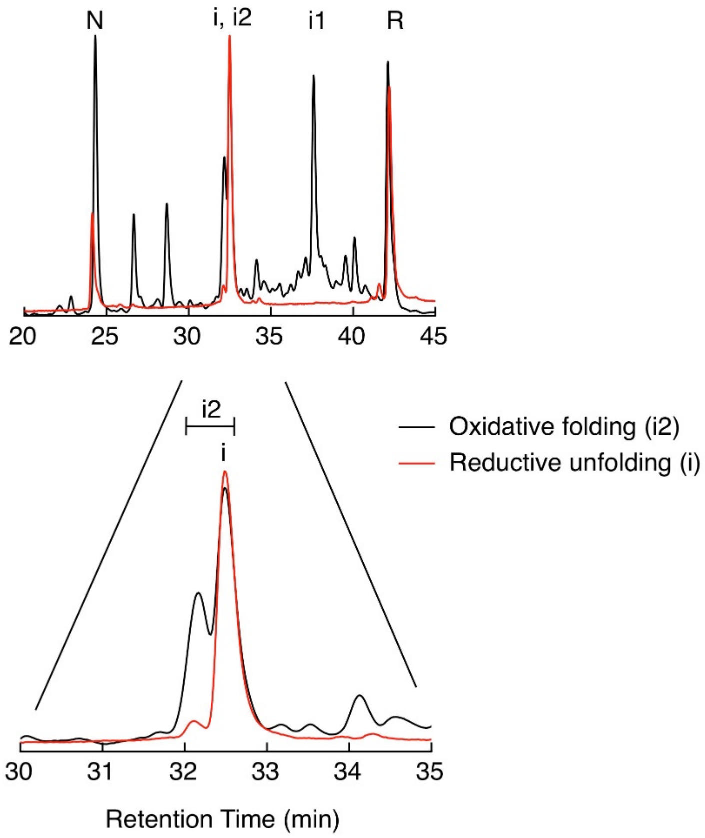


**Figure S5.** ***Comparison of RP-HPLC chromatographic profiles of TRIAP1 oxidative folding and reductive unfolding intermediates.*** RP-HPLC chromatographic profiles of acid-quenched intermediates of refolding reaction at 4 min, in the presence of 1 mM GSSG, 50 mM Tris.HCl, pH 8.4 (black line) and reductive unfolding quenched at 30 min in the presence of 32 mM TCEP in 0.1 M sodium acetate pH 5.0 (red line). Below is shown a close up of intermediates i and i2, where the heterogeneity of oxidative folding intermediate i2 is clearly observed.

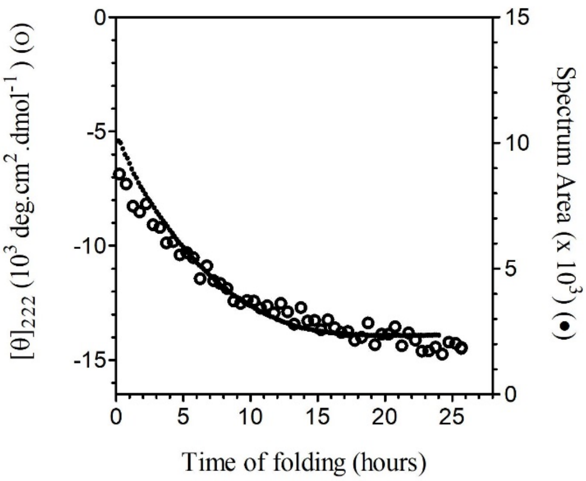


**Figure S6.** ***Secondary and tertiary structural changes upon oxidative folding of TRIAP1.*** The secondary and tertiary structure changes along folding time were monitored by far-UV CD and tryptophan fluorescence spectra measurements, respectively. The fully reduced/unfolded protein was allowed to refold in 0.1 M Tris-HCl pH 8.4 at 20 °C. Left Y axis represents the molar ellipticity changes at 222 nm over time. Right Y axis shows the changes in the tryptophan spectrum area as a function of folding time. Both signals were coupled along refolding. The experiment was performed twice, with similar results, a representative experiment is shown.

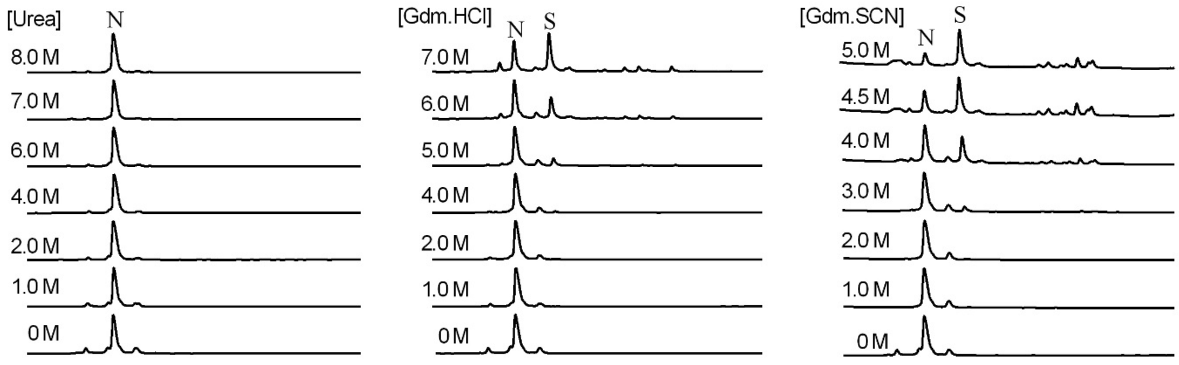


**Figure S7.** ***Disulfide scrambling of TRIAP1 under increasing concentrations of denaturants.*** The native form of TRIAP1 was incubated at 20 °C for 20 h in Tris.HCl (pH 8.4) containing 0.25 mM 2-mercaptoethanol as thiol initiator and the indicated concentration of denaturants. The samples were incubated in increasing concentrations of urea (left panel), Gdm.HCl (middle panel) and Gdm.SCN (right panel). The denatured samples were quenched with 1% TFA and analyzed by RP-HPLC. The native (N) and the major scrambled species (S) peaks are indicated.

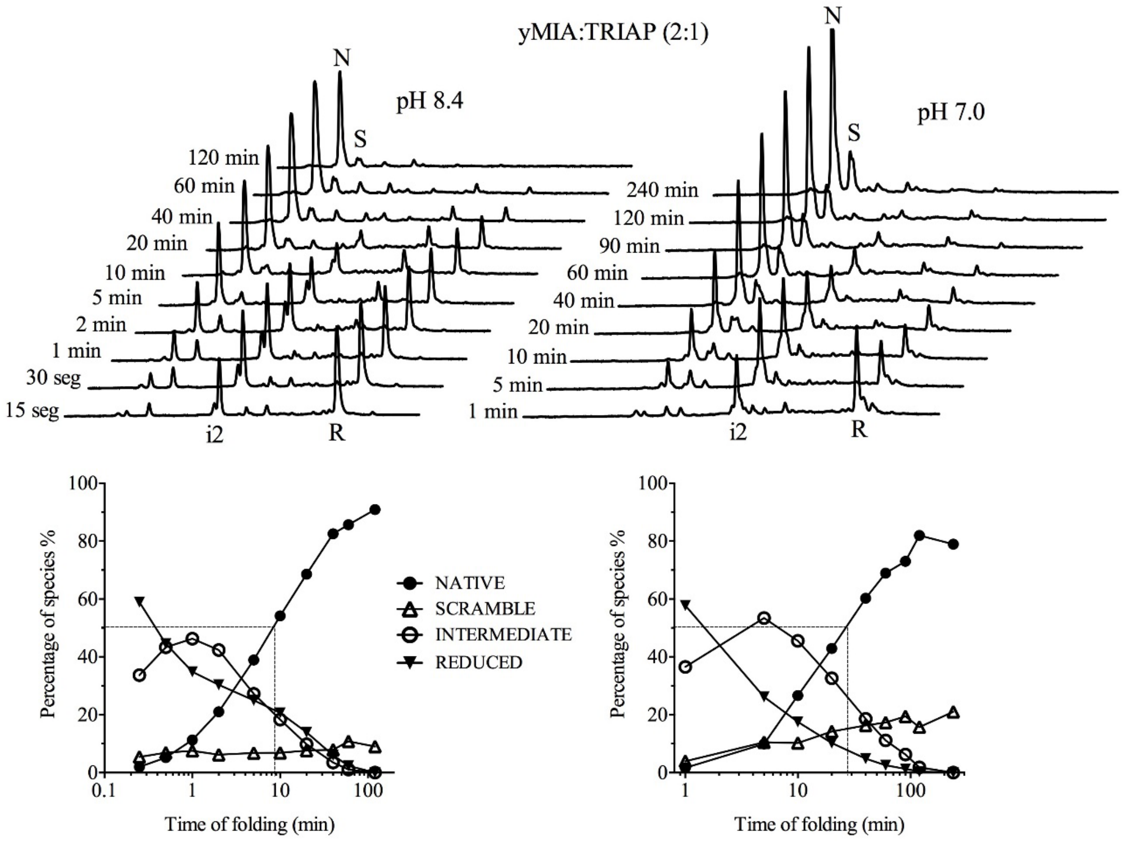


**Figure S8.** ***In-vitro oxidative folding of TRIAP1 catalyzed by yeast Mia40 (yMia40)***. GST-tagged yMia40 was added to TRIAP1 at a 2-fold molar excess in 50 mM Tris.HCl, 0.1 M NaCl, pHs: 8.4 (left panel) or 7.0 (right panel). The samples were acid-quenched at the indicated time points and subjected to RP-HPLC. The area under the curve of major RP-HPLC peaks were integrated and plotted as the percentage of species as a function of folding time (lower panel). The peaks integrated are: native (N), reduced (R), intermediate (i2) and scramble (s). Experimental results were consistent across three independent replicates. A representative experiment is shown.

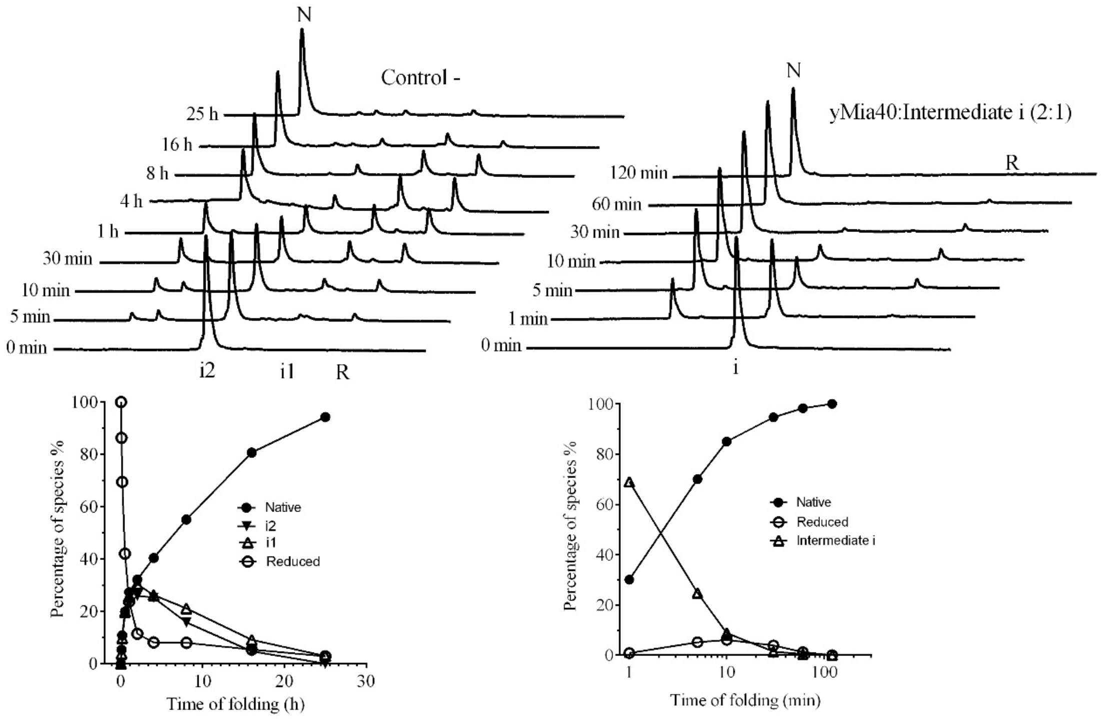


**Figure S9.** ***In-vitro oxidative folding of Cys18-Cys37 disulfide TRIAP1 intermediate (i) catalyzed by yeast Mia40 (yMia40).*** GST-tagged yMia40 was added to RP-HPLC purified intermediate Cys18-Cys37 TRIAP1 at a 2-fold molar excess in 50 mM Tris.HCl (pH 8.4), 0.1 M NaCl (right panel). As a control, a stop-go folding reaction of the intermediate i was performed in the absence of yMia40 in the same buffer condition at RT (left panel, Control -). The samples were acid-quenched at the indicated time points and subjected to RP-HPLC. The area under the curve of major RP-HPLC peaks were integrated and plotted as the percentage of species as a function of folding time (lower panel). The peaks integrated are: native (N), reduced (R), intermediates i, i1 and i2 (i1 and i2). The experiment was performed twice, with similar results, a representative experiment is shown.

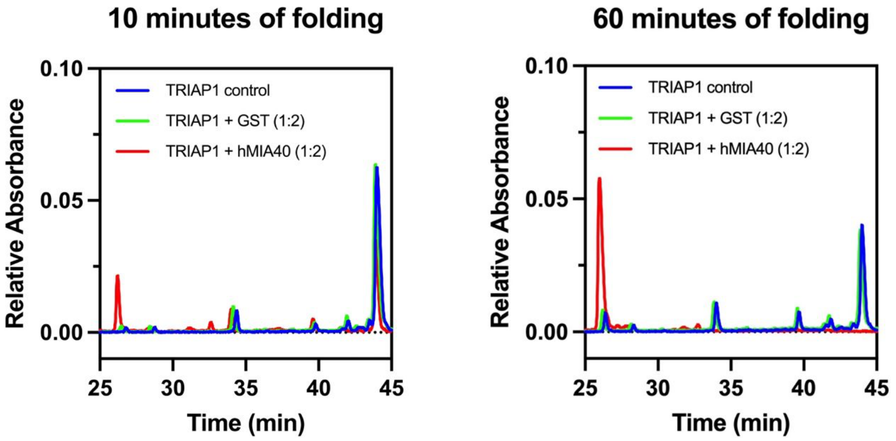


***Figure S10: The GST tag does not interfere with the in-vitro oxidative folding pathway of TRIAP1*** Reduced TRIAP1 is allowed to refold in the presence and in the absence of pure GST at a 1:2 TRIAP1:GST ratio, in 100 mM Tris.HCl pH 8.4. The reaction is then freezed by acidification with 1% of TFA at 10 min. (left panel) and 1 hour (right panel). Samples are loaded into a RP-HPLC connected to a C4 column (Phenomenex) and analyzed by acetonitrile 20- to 0 % gradient as described in the materials section of the manuscript. hMIA40 indicates GST-hMIA40 fusion protein
